# Exomer Is Part of a Hub Where Polarized Secretion and Ionic Stress Connect

**DOI:** 10.3389/fmicb.2021.708354

**Published:** 2021-07-19

**Authors:** Sandra Moro, Esteban Moscoso-Romero, Abhishek Poddar, Jose M. Mulet, Pilar Perez, Qian Chen, M.-Henar Valdivieso

**Affiliations:** ^1^Instituto de Biología Funcional y Genómica, Consejo Superior de Investigaciones Científicas, Salamanca, Spain; ^2^Departamento de Microbiología y Genética, Universidad de Salamanca, Salamanca, Spain; ^3^Department of Biological Sciences, University of Toledo, Toledo, OH, United States; ^4^Instituto de Biología Molecular y Celular de Plantas, Universitat Politècnica de València-Consejo Superior de Investigaciones Científicas, Valencia, Spain

**Keywords:** endosome, exomer, ionic stress, ion homeostasis, potassium, secretion, vesicle traffic, yeast

## Abstract

Plasma membrane and membranous organelles contribute to the physiology of the Eukaryotic cell by participating in vesicle trafficking and the maintenance of ion homeostasis. Exomer is a protein complex that facilitates vesicle transport from the *trans*-Golgi network to the plasma membrane, and its absence leads to the retention of a set of selected cargoes in this organelle. However, this retention does not explain all phenotypes observed in exomer mutants. The *Schizosaccharomyces pombe* exomer is composed of Cfr1 and Bch1, and *cfr1Δ* and *bch1Δ* were sensitive to high concentrations of potassium salts but not sorbitol, which showed sensitivity to ionic but not osmotic stress. Additionally, the activity of the plasma membrane ATPase was higher in exomer mutants than in the wild-type, pointing to membrane hyperpolarization, which caused an increase in intracellular K^+^ content and mild sensitivity to Na^+^, Ca^2+^, and the aminoglycoside antibiotic hygromycin B. Moreover, in response to K^+^ shock, the intracellular Ca^2+^ level of *cfr1Δ* cells increased significantly more than in the wild-type, likely due to the larger Ca^2+^ spikes in the mutant. Microscopy analyses showed a defective endosomal morphology in the mutants. This was accompanied by an increase in the intracellular pools of the K^+^ exporting P-type ATPase Cta3 and the plasma membrane Transient Receptor Potential (TRP)-like Ca^2+^ channel Pkd2, which were partially diverted from the *trans*-Golgi network to the prevacuolar endosome. Despite this, most Cta3 and Pkd2 were delivered to the plasma membrane at the cell growing sites, showing that their transport from the *trans*-Golgi network to the cell surface occurred in the absence of exomer. Nevertheless, shortly after gene expression in the presence of KCl, the polarized distribution of Cta3 and Pkd2 in the plasma membrane was disturbed in the mutants. Finally, the use of fluorescent probes suggested that the distribution and dynamics of association of some lipids to the plasma membrane in the presence of KCl were altered in the mutants. Thus, exomer participation in the response to K^+^ stress was multifaceted. These results supported the notion that exomer plays a general role in protein sorting at the *trans*-Golgi network and in polarized secretion, which is not always related to a function as a selective cargo adaptor.

## Introduction

Secretion and endocytosis are essential cellular processes that mediate protein trafficking between the cell interior and exterior. The secretory and endocytic pathways are composed of membranous compartments that communicate through the generation, movement, and fusion of coated vesicles (Palade, 1975; Behnia and Munro, 2005). Vesicular trafficking allows the transport of soluble cargoes and membrane components between compartments, contributing to the maintenance of their protein and lipid composition. Several protein coats and adaptors facilitate specific trafficking between compartments (Bonifacino and Lippincott-Schwartz, 2003; Paczkowski et al., 2015).

Exomer is a protein complex required for the transport of selected cargoes from the *trans-*Golgi network (TGN) to the plasma membrane (PM) that was first described in *Saccharomyces cerevisiae* (Sanchatjate and Schekman, 2006; Trautwein et al., 2006; Wang et al., 2006). It is composed of Chs5 and any two ChAPs (Chs5 and Arf1 binding Proteins; Chs6, Bud7, Bch1, and Bch2). Chs5 acts as a scaffold; Bch1 and Bud7 cooperate with the GTPase Arf1 to facilitate membrane bending, and Chs6 and Bch2 participate in cargo recognition (Sanchatjate and Schekman, 2006; Trautwein et al., 2006; Wang et al., 2006; Paczkowski et al., 2012, 2015; Paczkowski and Fromme, 2014; Huranova et al., 2016). Exomer cargoes are integral membrane proteins that localize at the PM of the sites of polarized growth—the mother–daughter neck, the small buds, and the shmoo tip. The best-characterized exomer cargo is the chitin synthase Chs3. In exomer mutants, Chs3 trafficking is blocked at the TGN; as a consequence, this enzyme does not reach the PM and cells are defective in chitin synthesis (Santos et al., 1997; Santos and Snyder, 1997; Sanchatjate and Schekman, 2006; Trautwein et al., 2006; Rockenbauch et al., 2012; Starr et al., 2012). Exomer mutants are also defective in cell fusion during mating due to the defect in Fus1 trafficking (Santos et al., 1997; Santos and Snyder, 2003; Barfield et al., 2009). Additional phenotypes reported for exomer mutants are altered random budding patterns, fast growth at a pH of 7.5, and sensitivity to ammonium, lithium, sodium, and hygromycin B (Santos and Snyder, 1997; Trautwein et al., 2006; Fell et al., 2011; Ritz et al., 2014). Anton et al. (2017) undertook an analysis of the potential cargo(es) that would be retained at the TGN in exomer mutants, resulting in their sensitivity to alkali metal cations. This study led to the conclusion that the sensitivity is explained, at least partially, by a defect in the localization of the Na^+^ extrusion pump Ena1. In exomer mutants, Ena1 reaches the PM of the mother cell but not that of the small buds. These results suggest that, although exomer facilitates the exit of certain proteins from the TGN, it is not always essential for this process. Additionally, this work underscored the relevance of exomer in polarized secretion (Valdivia et al., 2002; Bonifacino, 2014).

Exomer is present in Fungi and absent from Metazoa (Trautwein et al., 2006; Anton et al., 2018; Ramirez-Macias et al., 2018). All fungi bear one Chs5 homolog and one, two, or four ChAPs, with a Bch1 homolog always present. An analysis of the phenotypes associated with the absence of an exomer in yeasts with four ChAPs (*S. cerevisiae*), two ChAPs (*Kluyveromyces lactis* and *Candida albicans*), and one ChAP (*Ustilago maydis*) showed that the regulation of chitin synthesis by exomer was a late evolutionary acquisition (Anton et al., 2018). This function arose concomitant with the appearance of multiple ChAPs and their specialization as cargo adaptors. This fact explains the presence of exomer components in *Schizosaccharomyces pombe*, a yeast without detectable chitin (Horisberger and Rosset, 1977; Arellano et al., 2000).

In *S. pombe*, Cfr1 is a Chs5 homolog that, together with a single ChAP (Bch1), constitutes a functional exomer (Cartagena-Lirola et al., 2006; Martin-Garcia et al., 2011; Hoya et al., 2017). Exomer mutants exhibit mild defects in cell wall synthesis, fusion during mating, and growth under different sources of stress. In particular, exomer mutants are sensitive to KCl, a condition that leads to defects in septum formation (Hoya et al., 2017). Analysis of the localization of transmembrane proteins that are involved in these processes showed that none of them was retained at the TGN or mislocalized. Nevertheless, the simultaneous deletion of exomer and Apm1 produced severe defects in the traffic through and the morphology of the TGN/early endosomes, and simultaneous deletion of exomer and Gga22 produced defects in the traffic through and the morphology of the prevacuolar/late endosome (PVE). These results led to the conclusion that exomer was not a classical cargo adaptor but cooperated with clathrin adaptors in protein sorting in the TGN, and in the maintenance of TGN, and PVE integrity (Hoya et al., 2017). Since membranous cell compartments participate in vesicle trafficking and ionic homeostasis (Scott and Gruenberg, 2011), characterizing the participation of exomer in KCl tolerance will add to understanding of the relationship between both processes.

K^+^ is the most abundant cation in cells, and cells have developed import and export systems to maintain the intracellular potassium concentration into a range whose alteration would produce cell damage and alterations in physiology (Mulet et al., 2013). The coordinated and well-regulated action of K^+^ influx and efflux transporters guarantees K^+^ homeostasis (Calero and Ramos, 2003; Arino et al., 2014, 2019). *In S. pombe*, Trk1 and Trk2 are the main influx transporters. Trk1 and Trk2 are high-affinity transporters whose single deletion leads to mild sensitivity to NaCl but not to limiting K^+^ (Lichtenberg-Frate et al., 1996a,b; Balcells et al., 1999; Calero et al., 2000). Conversely, deleting both *trk1*^+^ and *trk2*^+^ lead to severe growth reduction when K^+^ is scarce, demonstrating that the presence of one of the transporters is sufficient to maintain K^+^ homeostasis (Calero et al., 2000, 2004). Additionally, *trk1*Δ *trk2*Δ is sensitive to Na^+^, hygromycin B, and low pH (Calero et al., 2004). *trk1*Δ *trk2*Δ grows well at K^+^ [20 mM] and higher and transports Rb^+^ with low affinity, suggesting that other transporters, which may be non-specific, uptake the ion under these conditions. Some results suggest that this putative transporter might be regulated by the intracellular potassium concentration. An alternative would be an ectopic K^+^ entrance stimulated by alterations in internal pH and/or alterations in membrane potential, as described in budding yeast (Madrid et al., 1998). Regarding K^+^ efflux, *S. pombe* Cta3 is a specific efflux P-type ATPase that mediates the export of K^+^ but not Na^+^ (Benito et al., 2002). The *S. pombe* genome does not bear additional genes with sequence homology to Trk1 and/or Trk2 but does bear homologs to other *S. cerevisiae* transporters (Wood et al., 2002; Ramos et al., 2011; Wood et al., 2012). The *S. cerevisiae NHA1* homolog —*nhe1*^+^/*sod1*^+^— is a specific Na^+^ transporter whose deletion results in sensitivity to NaCl but not to KCl (Balcells et al., 1997; Kinclova et al., 2002). *kha1*^+^ is a homolog to the *KHA1* K^+^/H^+^ antiporter, which is potentially involved in the regulation of K^+^ and pH homeostasis in the Golgi apparatus, and whose deletion produces K^+^ accumulation (Ramirez et al., 1998; Arino et al., 2014; Yenush, 2016). The participation of SpKha1 in K^+^ homeostasis has not yet been reported.

While deleting genes involved in K^+^ homeostasis leads to growth defects in media with limiting K^+^ (Calero and Ramos, 2003), sensitivity to K^+^ is an infrequent phenotype. This phenotype has been reported for the *S. pombe pzh1*Δ mutant, which is defective in a PPZ-like phosphatase that regulates Na^+^ influx in a Sod2- and Trk1-independent fashion (Balcells et al., 1997, 1999), and in the *S. cerevisiae ena1-4*Δ *kha1*Δ mutant that is defective in K^+^ efflux systems (Benito et al., 2002). Therefore, analyzing the function of the *S. pombe* exomer in the tolerance to high external K^+^ will help to understand the mechanisms underlying this tolerance and the participation of exomer in the process. Additionally, these studies will shed light on the evolutionarily conserved aspects of exomer function in response to ionic stress. Finally, since K^+^ homeostasis is related to drug and antibiotic sensitivity, regulation of blood pressure, and crop resistance to salinity (Alao et al., 2015; Isayenkov and Maathuis, 2019; Nomura et al., 2019), this information is valuable from a therapeutic and economic point of view. In this work, we undertook a detailed characterization of the sensitivity of exomer mutants to KCl and the functional relationship between this complex and proteins involved in K^+^ and Ca^2+^ homeostasis. Our results showed that exomer mutants have a plethora of small defects in the trafficking of ion transporters, in ion homeostasis, and in the distribution of lipids in the PM suggesting that their sensitivity to K^+^ is multifaceted and not due to the mistargeting of a single protein. The results point to a role of exomer in the coordination of multiple processes whose simultaneous alteration leads to sensitivity to KCl.

## Materials and Methods

### Strains and Growth Conditions

All general growth conditions and yeast manipulations were performed as previously described (Moreno et al., 1991; Forsburg and Rhind, 2006). The relevant genotypes and the source of the utilized strains are listed in Supplementary Table 1. All experiments were performed with cells from cultures growing exponentially. Normally, cells were cultured in YES (Yeast Extract with Supplements; 0.5% yeast extract, 3% glucose, 225 mg/l adenine sulfate, histidine, leucine, uracil, and lysine). When required, cells were grown in EMM2 (Edinburgh Minimal Medium) (Moreno et al., 1991) with supplements. For drop-test analyses to assess sensitivity to ionic stress, 3 × 10^4^ cells and serial 1:4 dilutions were inoculated on YES plates supplemented or not with different salts and incubated at 32°C for 3 days. When the assays were performed on minimal EMM2 plates, KNO_3_ was preferred to KCl for technical reasons, and the plates were incubated for 5 days. Geneticin (G418, Formedium), hygromycin B (Formedium), and nourseothricin (Werner BioAgents) were used at 120, 400, and 50 μg/ml, respectively.

### Genetic Methods

Molecular and genetic manipulations were performed according to Sambrook and Russell (2001). Gene deletions and tagged proteins were normally generated by transforming a *pku70*Δ strain (Fennessy et al., 2014) with polymerase chain reaction (PCR)-generated modules, as previously described (Bähler et al., 1998). The resulting transformants were backcrossed to reintroduce the *pku70*^+^ allele. A GFP-Trk1 fusion protein expressed from the *nda2*^+^ promoter, which yielded a constitutive 5-fold overexpression (PomBase^1^ ; Wood et al., 2012; Lock et al., 2018a,b), was constructed by fusion PCR (Cha-Aim et al., 2012). The construct bore (5′ to 3′) a 450-bp DNA fragment upstream from the *trk1*^+^ 5′ untranslated (UTR) region, the *HPHMX6* hygromycin B resistance gene, used as a selection marker, a 571-bp DNA fragment corresponding to the *nda2*^+^ 5′ UTR, the GFP, and the first 440 bases from the *trk1*^+^ ORF. This fragment was used to transform yeast cells such that it was integrated into the *trk1*^+^ locus. A *pkd2*^+^ gene under the control of the *cta3*^+^ promoter was constructed by fusion PCR of the following DNA fragments (5′ to 3′); 1,467 bp corresponding to the 5′ *cta3*^+^ UTR and upstream sequences, followed by the *pkd2*^+^ ORF fused upstream from the GFP. This cassette was cloned as a *Pst*I/*Sma*I fragment upstream from the *nmt1*^+^ terminator in the pINTH1 vector (Fennessy et al., 2014). The plasmid was digested with *Not*I, and the P*cta3*^+^:pkd2-GFP:T*nmt1*^+^ cassette was used to transform the hph.171 K strain (Fennessy et al., 2014, YGRC #FY23692). This strategy was used to maintain an intact *pkd2*^+^ locus, allowing Pkd2 production in the absence of KCl. The GAP1(PH) PI(4,5)P2-binding probe was PCR-amplified from plasmid pEGFP-C1-PH-GAP1IP4BP (#20200 Addgene; Hammond et al., 2009), ligated to the C-terminal end of the GFP and cloned under the control of the *nda2*^+^ promoter and terminator into pINTH1. The construct was transformed into the hph.171K strain. The accuracy of all constructions was assessed by DNA sequencing and correct integration by PCR. Depending on technical requirements, *cfr1Δ* or *bch1Δ*, which have the same phenotypes (Hoya et al., 2017), were used as exomer mutants. Different genetic traits were combined either by genetic crosses with *cfr1Δ* and posterior selection of the traits of interest by random spore analysis (Forsburg and Rhind, 2006), or by transformation with cassettes for *bch1Δ* deletion.

### Microscopy

For conventional fluorescence microscopy, a Nikon Eclipse 90i microscope (100x objective; numerical aperture 1.45), equipped with a Hamamatsu ORCA ER camera, was used; images were captured using MetaMorph Premier. To obtain images with better resolution, an Olympus IX71 microscope (objectives 100 × with numerical aperture 1.4, and 60x with numerical aperture 1.42) equipped with a personal DeltaVision system and a Photometrics CoolSnap HQ2 monochrome camera, was used; stacks of three Z-series sections corresponding to the cell middle were acquired at 0.2 μm intervals and images were processed using deconvolution Softworx DV software (Applied Precision). For confocal live-cell imaging, a spinning-disk Olympus IX-81 microscope equipped with a confocal CSUX1-A1 module (Yokogawa) and an Evolve (Photometrics) camera was used; images were acquired using Metamorph software. Typically, to analyze protein colocalization, stacks of three 0.2 μm Z-sections of the cell middle were acquired, and the central plane of each stack was analyzed; the captured images were saved as 16-bit images, filtered with Fiji software (ImageJ, National Institutes of Health, United States), and quantified manually. The micrographs to produce the 3-dimensional reconstructions shown in the right panel of Figure 4F, and the micrographs in Figure 7B were captured using an ANDOR Dragonfly Spinning disk Nikon Ti2-E microscope equipped with a sCMOS Sona 4.2B-11 camera. Images were acquired using ANDOR Fusion software and processed with Fiji and Imaris (Oxford Instruments). To estimate dot intensity, the maximum intensity of a straight line traced through each dot was measured. To estimate the size of dots, an elliptical region of interest (ROI) was drawn, and its intensity was measured using Fiji. To estimate D4H intensity, a line was drawn across the plasma membrane, and the maximal value was scored. The following lipid-binding probes were used: mCherry-FYVE(EEA1)—specific for phosphatidylinositol-3-phosphate (PI3P)—was used as a marker for the PVE, and mCherry-FAPP1(PH)—specific for PI4P—was used as a marker for the TGN (Hoya et al., 2017; Yanguas et al., 2019). GFP-GAP1(PH) and mCherry-D4H were used to detect the PM distribution of PI(4,5)P2 and sterols, respectively (Hammond et al., 2009; Marek et al., 2020). Intracellular calcium concentration and calcium transients were determined using GCaMP6s (Poddar et al., 2021) as follows. For time-lapse microscopy, 30 μl of the exponentially growing cells was spotted directly onto a coverslip (#1.5) bottomed Petri dish (Cellvis, United States). The coverslip was pre-coated with 50 μl of 50 μg/ml lectin (Sigma, L2380) and allowed to dry overnight at 4°C. After incubation for 12 min at room temperature to allow the cells to attach, 2 ml of YES plus 0.6 M KCl was added to the dish. When chelation of calcium was required, 2 ml of YES plus 0.6 M KCl and 2 mM EGTA was used. The time-lapse imaging started exactly 6 min after the addition of YES plus KCl to the dish. For the time-lapse microscopy, we employed a spinning-disk confocal microscope. The microscope was an Olympus IX-71 unit equipped with a CSU-X1 spinning-disk unit (Yokogawa, Japan). The motorized stage (ASI, United States) included a Piezo Z Top plate for acquiring Z-series. The images were captured on an EMCCD camera (IXON-897, Andor) controlled by iQ3.0 (Andor). Solid-state lasers of 488 and 561 nm were used in fluorescence microscopy at a power of no more than 2.5 mW. We used a 60x objective lens (Olympus, Plan Apochromat, NA = 1.40). A *z*-series of eight slices at a spacing of 1 μm was captured at each time point throughout the time-lapse series. The room temperature was maintained at 22 ± 2°C. To further minimize environmental variations, we imaged both control and experimental groups in a randomized order on the same day. The intracellular fluorescence of GCaMP6s-expressing cells was quantified using ImageJ. Average intensity projections of the *Z*-series were used for quantitative image analysis. For each experimental group, 20 randomly selected cells were quantified. Average fluorescence intensities of 2–3 cytoplasmic regions, squares measuring approximately 1 μm^2^, was measured for each cell. All results were represented and statistically analyzed with Graphpad Prism software (GraphPad Software Inc., United States). The specific test used after ANOVA (ANalysis Of VAriance) for each analysis is specified in the corresponding figure legend. Electron microscopy was performed as described (De Leon et al., 2013). Cells were fixed in glutaraldehyde (TAAB) and stained with 2% potassium permanganate, dehydrated in ethanol, embedded in TAAB Spurr’s Resin, and stained with uranyl acetate. Ultrathin sections were produced using a Leica EM UC7 microtome and examined using a Tecnai Spirit Twin 120 kV transmission electron microscope, equipped with a LaB6 filament and a CCD Wide-angle lateral and bottom camera.

### Protein Methods

Trichloroacetic acid (TCA) protein precipitation from cell extracts and western blot analysis were performed as described (Yanguas et al., 2019). Cells growing exponentially in 30 ml YES were collected by centrifugation (900 × *g*), washed with 1 ml of cold 20% TCA, and resuspended in 50 μl of the same solution. Five hundred μl of glass beads (Braun Biotech International) were added, and the cells were broken in a cold Fast Prep FP120 (Savant Bio101), with 5-min incubations on ice between pulses. Next, 400 μl cold 5% TCA was added to the tube, which was vortexed to wash the beads. Cell extracts were then transferred to another tube and centrifuged for 10 min at 4°C. The pellets were resuspended in 2% sodium dodecyl sulfate (SDS)/0.3 M Tris base. The protein concentration was determined using a Bradford protein assay reagent (Bio-Rad). Equal protein amounts were boiled in the presence of the Laemmli sample buffer (50 mM Tris-HCl, pH 6.8; 1% SDS; 143 mM β-mercaptoethanol; and 10% glycerol) for 5 min. Samples were subjected to polyacrylamide gel electrophoresis (PAGE), transferred to polyvinylidene difluoride (PVDF) membranes, and incubated in blocking buffer (5% Nestlé non-fat dried milk in TBST: 0.25% Tris, pH 7.6; 0.9% NaCl; and 0.25% Tween 20) for 1 h. Primary antibodies were monoclonal anti-GFP (JL8, BD Living Colors; 1:3,000) and anti-tubulin (clone B-5-1-2; 1:10,000), and polyclonal anti-Pma1 (Goossens et al., 2000; 1:10,000). The secondary antibodies were horseradish peroxidase-conjugated anti-mouse (Bio-Rad; 1:10,000) and anti-rabbit (clone RG-96, Sigma; 1:10,000). The chemiluminescent signal was detected using the Western Bright ECL detection kit (Advansta) and either X-ray films (Agfa) or a Vilber Fusion FX system (Vilber GmbH).

### Biochemical Methods

To estimate the intracellular potassium concentration, cells were grown in YES medium to an absorbance of 0.6–0.7 at 660 nm, centrifuged for 5 min at 1900 × *g*, resuspended at the same concentration in fresh YES medium, and incubated at 30°C for 90 min. Aliquots were taken, centrifuged in plastic tubes for 5 min at 700 × *g* and 4°C, and washed twice with 10 ml of ice-cold solution of 20 mM MgCl_2_. The cell pellets were resuspended in 0.5 ml 20 mM MgCl_2_. Ions were extracted by heating the cells for 15 min at 95°C. After centrifugation, aliquots of the supernatant were analyzed with an atomic absorption spectrometer (SensAA) in ‘flame emission’ mode as previously described (Rios et al., 2013). Plasma membrane ATPase activity was performed by measuring ATP consumption by determining the amount of inorganic phosphate with ammonium molybdate, as described (Perez-Valle et al., 2010).

## Results

### Exomer Mutants Are Sensitive to K^+^ and Accumulate K^+^

**FIGURE 1:**
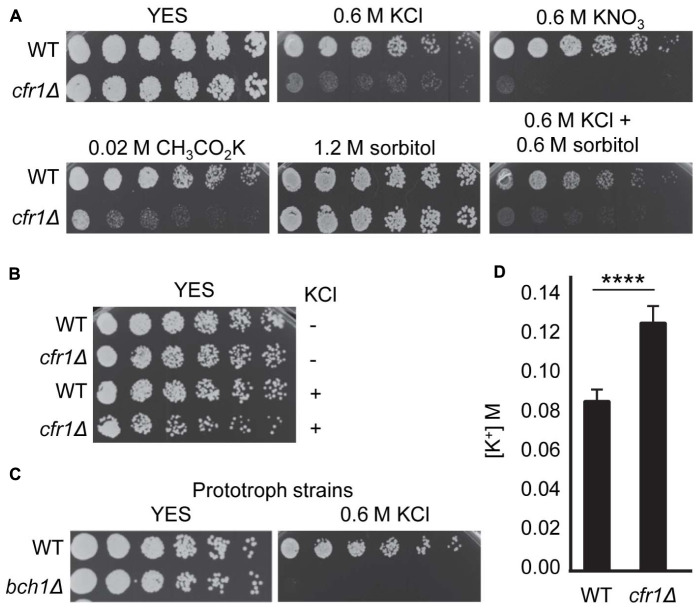
Exomer mutants are sensitive to K^+^ and hyperaccumulate this ion. **(A)** Cells from the wild-type (WT) and *cfr1Δ* strains were spotted on YES (rich medium) and YES supplemented with the indicated compounds. **(B)** Cells from the WT and *cfr1Δ* strains were incubated in YES medium (KCl, –) and in YES medium with 0.6 M KCl (KCl, +) for 16 h, diluted, spotted on YES plates, and incubated at 32°C for 2 days. **(C)** Cells from prototroph WT and *bch1Δ* strains were spotted on YES and YES with 0.6 M KCl. **(D)** Intracellular K^+^ concentration (M) in the WT and *cfr1Δ* strains. For each value, the mean of three independent experiments, standard deviation, and statistical significance of the difference, determined using the *t*-test are shown (****p < 0.0001).

To gain information about the role of exomer in stress response, we characterized potassium sensitivity in mutants. First, we determined whether *cfr1Δ* sensitivity to high KCl concentrations was due to an inability to grow under ionic or osmotic stress. To do so, we analyzed *cfr1Δ* sensitivity to several potassium salts at different concentrations, which depended on the concentration that inhibited the growth of the WT, and to sorbitol (Figure 1A). *cfr1Δ* exhibited growth defects in the presence of 0.6 M potassium chloride (KCl), 0.6 M potassium nitrate (KNO_3_), and 0.02 M potassium acetate (CH_3_CO_2_K). In contrast, the mutant grew well in the presence of 1.2 M sorbitol, a medium with similar osmolarity to 0.6 M KCl. These results showed that *cfr1Δ* was sensitive to KCl because of a sensitivity to K^+^ but not to chloride or high osmolarity. Since exomer mutants exhibit mild cell wall defects (Hoya et al., 2017), we wondered whether the presence of K^+^ enhanced these defects leading to cells lysis. To address this question, we inoculated wild-type (WT) and *cfr1Δ* cells on plates supplemented with 0.6 M KCl plus 0.6 M sorbitol. We found that sorbitol did not improve growth in the presence of KCl (Figure 1A). Furthermore, we incubated WT and *cfr1Δ* cells in liquid YES medium with and without 0.6 M KCl for 16 h, diluted the cultures, and plated them on YES plates. Under these conditions, most *cfr1Δ* cells treated with KCl produced colonies in the absence of osmotic support (Figure 1B), as did WT cells and untreated *cfr1Δ* cells, which negated the hypothesis that sensitivity to potassium was the consequence of lysis because of alterations in the cell wall.

The transport of ions and amino acids influence each other (Brown and Sepulveda, 1985; Castagna et al., 1998), and budding yeast exomer mutants are defective in tryptophan uptake (Anton et al., 2021). Additionally, *cfr1Δ* and *bch1Δ* are defective in trafficking to the PVE/vacuoles, and these organelles are important for amino acid homeostasis (Hoya et al., 2017; Lawrence and Zoncu, 2019). Consequently, to understand whether the presence of K^+^ produced a defect in amino acid uptake/homeostasis that might be enhanced in exomer mutants and might result in growth defects of auxotrophic strains, we analyzed the growth of prototrophic WT and *bch1Δ* strains on YES plates with 0.6 M KCl. We found that the mutant was sensitive under these conditions (Figure 1C).

Finally, to investigate whether exomer mutants exhibited altered K^+^ homeostasis, we determined the intracellular K^+^ content of WT and *cfr1Δ* cells. The results showed that this content was significantly higher in the exomer mutant than in the WT (Figure 1D), data that confirmed that exomer plays a role in the regulation of K^+^ homeostasis.

### Exomer Mutants Interact Genetically With Mutants in K^+^ Transporters and Channels

**FIGURE 2:**
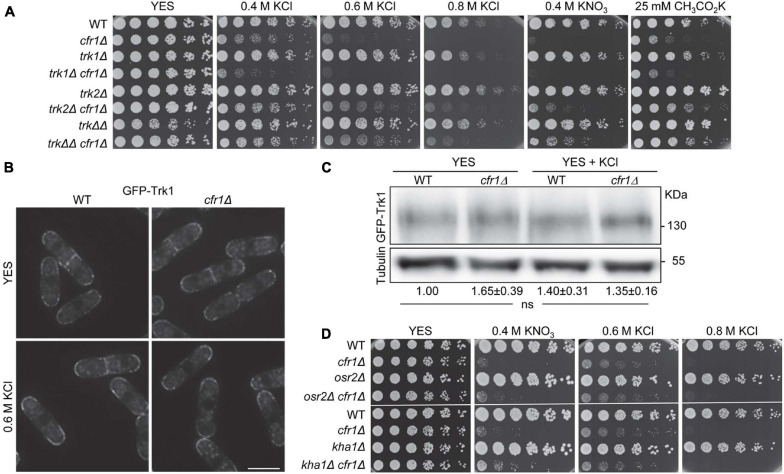
Relationship between exomer and Trk transporters in the presence of potassium salts. **(A)** Cells from the indicated strains were spotted on YES (rich medium) and YES supplemented with the indicated concentrations of potassium salts. WT, wild-type; *trkΔΔ*, *trk1Δ trk2Δ*. **(B)** DeltaVision images (SUM projections) of the WT and *cfr1Δ* cells bearing GFP-Trk1 and growing exponentially in YES and YES with 0.6 M KCl. Bar, 10 μm. **(C)** Cell extracts from the indicated strains containing GFP-Trk1, and incubated in YES and YES with 0.6 M KCl, were subjected to SDS-PAGE and immunoblotted with anti-GFP (upper panel) and anti-tubulin (lower panel; loading control) antibodies. The relative position of molecular weight markers is indicated on the right (KDa). The intensity of each GFP-Trk1 band was relativized to the value for the corresponding tubulin band, and all the values were relativized to the value for the WT grown in YES. For each value, the mean of three independent experiments, standard deviation, and statistical significance of the difference, determined using the Tukey’s multiple comparisons test are shown (ns, non-significant). **(D)** Cells from the indicated strains were spotted on YES (rich medium) and YES supplemented with the indicated concentrations of potassium salts.

Since exomer facilitates the trafficking of transmembrane proteins from the TGN to the PM, we investigated whether it was involved in the trafficking of a K^+^ transporter/channel. The best-characterized *S. pombe* potassium transporters, *trk1*^+^ and *trk2*^+^, exhibit growth defects under K^+^ limiting conditions (Calero et al., 2000, 2004). Nonetheless, we compared the growth of *trk1Δ* and *trk2Δ* with that of *cfr1Δ* in the presence of high K^+^ concentrations. Additionally, we constructed double and triple mutants to determine whether *cfr1*^+^ acts in the same functional pathways as *trk1*^+^ and/or *trk2*^+^. The results showed that while *cfr1Δ* was sensitive to potassium salts, neither *trk1Δ*, *trk2Δ* nor *trk1Δ trk2Δ* (denoted by *trkΔΔ* in the figure) exhibited sensitivity (Figure 2A). Regarding double mutants, *trk1Δ cfr1Δ* was more sensitive than *cfr1Δ*, indicating that both genes cooperated and acted in parallel rather than in linear pathways related to K^+^ sensitivity. In contrast, *trk2Δ* deletion partially suppressed the growth defect of *cfr1Δ* in the presence of K^+^ salts, indicating that both genes played opposing roles. The phenotype of *trk1Δ trk2Δ cfr1Δ* (*trkΔΔ cfr1Δ* in the figure) was similar to that of *trk2Δ cfr1Δ*. These results showed that the relationship between *trk1*^+^, *trk2^+^*, and *cfr1*^+^ in terms of potassium sensitivity was complex.

The phenotype of *cfr1Δ* was different from that of *trk1Δ* and *trk2Δ*, which *a priori* negated the hypothesis that *cfr1Δ* sensitivity to K^+^ was due to Trk1 and/or Trk2 retention in the TGN, which might enhance K^+^ uptake by other transporter(s) producing the hyperaccumulation of this ion. Nevertheless, to obtain direct information about this issue, we fused Trk1 and Trk2 to GFP to determine their localization. GFP-Trk1 localized at the cell surface of the cell growing sites (cell poles and equator) in both, WT and *cfr1Δ* cells (Figure 2B), confirming proper Trk1 sorting in the absence of exomer. Microscopy and western blotting showed that the amount of GFP-Trk1 was similar in both strains (Figures 2B,C). For unknown reasons, neither Trk2-GFP nor GFP-Trk2 produced a fluorescent signal. Nevertheless, the fact that *trk2Δ* partially suppressed *cfr1Δ* sensitivity indicated that the reason for this sensitivity was not the lack of Trk2 at the cell surface. In fact, this result indicated that Trk2 might mediate K^+^ uptake in *cfr1Δ*, at least partially.

To determine the involvement of exomer in the regulation of other potential K^+^ transporters, we analyzed the phenotype of *osr2Δ* (annotated in PomBase as a “Potassium channel, β subunit” ortholog of human KCNAB1-3, the β beta regulatory subunits of the K^+^ voltage-gated channel subfamily A), and the phenotype of *kha1Δ* (annotated as a “Plasma membrane K^+^ ion/proton antiporter”; ortholog of the *Saccharomyces cerevisiae KHA1* putative K^+^/H^+^ antiporter). We also analyzed the genetic interaction between *osr2*^+^, *kha1*^+^, and *cfr1*^+^. We found that neither *osr2Δ* nor *kha1Δ* was sensitive to K^+^ salts and that the phenotype of the corresponding double mutant was similar to that of the single *cfr1Δ* mutant (Figure 2D). Finally, we analyzed the growth of the strains from the *S. pombe* Genome-wide Deletion Mutant Library (Bioneer, United States) deleted in genes annotated as ion transporters/channels in the presence of 1 M KCl. None of the strains was as sensitive as *cfr1Δ* (Supplementary Figure 1).

In summary, all these results indicated that the sensitivity of exomer mutants to potassium salts was not the consequence of a mis-sorting of these proteins, at least not exclusively.

### Plasma Membrane Hyperpolarization in Exomer Mutants

**FIGURE 3:**
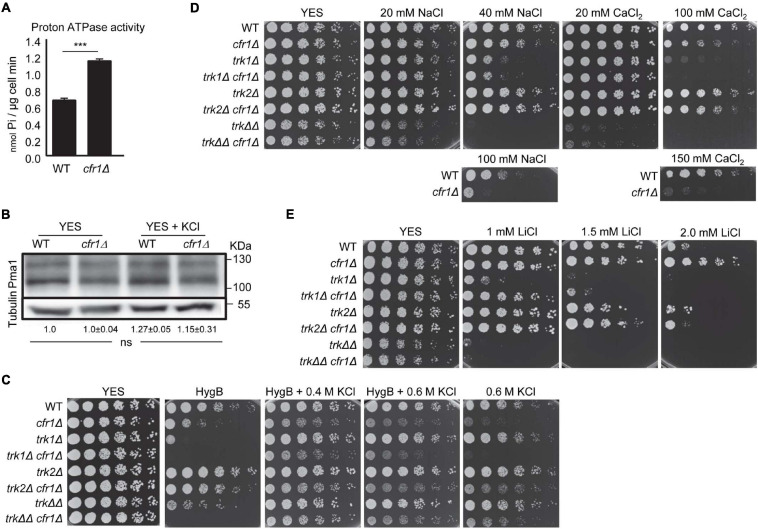
Analysis of membrane polarization in exomer mutants. **(A)** Measurement of ATPase activity (nmol Pi/μg cell minute) in the wild-type (WT) and *cfr1Δ* extracts. For each value, the mean of three independent experiments, standard deviation, and statistical significance of the difference, determined using the *t*-test are shown (***p < 0.001). **(B)** Cell extracts from the WT and *cfr1Δ* strains, incubated in YES and YES with 0.6 M KCl, were subjected to SDS-PAGE and immunoblotted with polyclonal anti-*S. cerevisiae* Pma1 (upper panel) and anti-tubulin (lower panel; loading control) antibodies. The relative position of molecular weight markers is indicated on the right (KDa). The intensity of each Pma1 band was relativized to the value for the corresponding tubulin band, and all the values were relativized to the value for the WT grown in YES. For each value, the mean of three independent experiments, standard deviation, and statistical significance of the difference, determined using the Tukey’s multiple comparisons test are shown (ns, non-significant). **(C)** Cells from the indicated strains were spotted on YES (rich medium) and YES supplemented with 2 μg/ml hygromycin B (HygB), 2 μg/ml hygromycin B with KCl, and KCl without hygromycin B. WT, wild-type; *trkΔΔ*, *trk1Δ trk2Δ*. **(D)** The same experimental details as in **(C)**, but the cells were spotted on YES plates supplemented with different amounts of NaCl and CaCl_2_. The lowest panels show the growth of the WT and *cfr1Δ* on plates supplemented with high concentrations of the same compounds. **(E)** The same experimental details as in **(C)**, but the cells were spotted on YES plates supplemented with different amounts of LiCl.

High ATPase activity extrudes protons at a high rate, leading to PM hyperpolarization, which induces the uptake of ions (K^+^, Na^+^, Li^+^, Ca^2+^) and some toxic compounds, such as hygromycin B (Madrid et al., 1998; Mulet et al., 1999; Calero et al., 2000). Therefore, to understand whether K^+^ accumulation in exomer mutants was related to high ATPase activity, we measured the H^+^ ATPase activity in WT and *cfr1Δ* strains. We found that this activity was significantly higher in the mutant (Figure 3A). According to western blotting, the increased activity in the mutant was not the consequence of increased amounts of Pma1 (Figure 3B).

To confirm that this increase in ATPase activity had an effect on the physiology of the mutant cells and to continue with the characterization of K^+^ sensitivity, we investigated whether PM was hyperpolarized in exomer mutants. Membrane hyperpolarization and intracellular K^+^ retention under some conditions have been reported in *trk1Δ trk2Δ*. In agreement, we compared the sensitivity to hygromycin B of single mutants lacking *cfr1*^+^, *trk1*^+^, *trk2*^+^ with that of double and triple mutants. The results are shown in the Figure 3, where *trkΔΔ* denotes *trk1Δ trk2Δ* and *trkΔΔ cfr1Δ* denotes *trk1Δ trk2Δ cfr1Δ*. *cfr1Δ* was partially sensitive to hygromycin B and deleting *cfr1*^+^ enhanced the sensitivity of *trk1Δ*, *trk2Δ*, and *trk1Δ trk2Δ* strains (Figure 3C). Hygromycin B sensitivity, together with the fact that supplementing the medium with 0.4 M KCl improved growth in the presence of the antibiotic, agreed with membrane hyperpolarization. Additionally, the antibiotic improved the growth of *cfr1*Δ and *trk1*Δ *cfr1*Δ in the presence of high KCl concentrations. Next, we analyzed growth in the presence of other ions (Figure 3D). The results showed that *trk1Δ* was partially sensitive to 40 mM NaCl and sensitive to 100 mM CaCl_2_; *trk2Δ* grew well under all conditions, and *trk1Δ trk2Δ* was the most sensitive of all the strains. *cfr1Δ* was only sensitive to very high Na^+^ and Ca^2+^ concentrations (lower panels in Figure 3D) and introducing the *cfr1Δ* mutation did not enhance the sensitivity of any *trkΔ* mutant. Regarding growth on lithium-supplemented plates, *trk1Δ* was partially sensitive to 1.0 mM LiCl and *trk1Δ trk2Δ* was very sensitive (Figure 3E). In contrast, *cfr1Δ* was resistant even to 2.0 mM LiCl, a concentration that reduced the growth of the WT, and *cfr1*^+^ deletion improved *trk1Δ* growth at 1.0 and 1.5 mM LiCl.

In summary, enhancement of the proton ATPase activity, *cfr1Δ* sensitivity to hygromycin B, and its suppression by 0.4 M KCl strongly supported the hypothesis that PM was hyperpolarized in exomer mutants. Nevertheless, the mild sensitivity to NaCl and the resistance to LiCl suggested that this hyperpolarization would not be very strong and might not explain potassium sensitivity in exomer mutants by itself.

### Distribution of the K^+^ Extrusion Pump Cta3 Is Abnormal in Exomer Mutants

**FIGURE 4:**
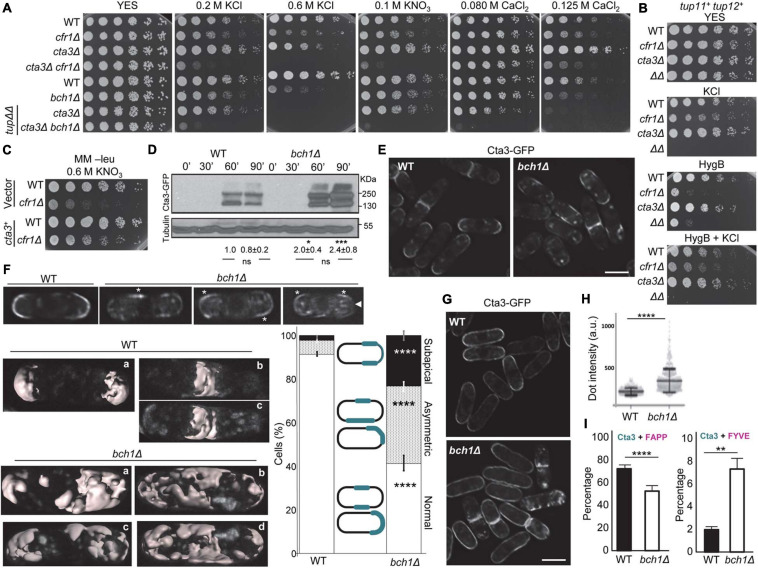
Relationship between exomer and the K^+^-exporting pump Cta3. **(A)** Cells from the indicated strains were spotted on YES (rich medium) and YES supplemented with the indicated compounds. *tupΔΔ* denoted that the *cta3Δ* and *cta3Δ bch1Δ* strains spotted at the bottom of the plate bore *tup11Δ* and *tup12Δ* deletions. **(B)** The same experimental details as in **(A)**, but the cells were inoculated on YES and YES supplemented with 0.4 M KCl, 2.0 μg/ml hygromycin B (HygB), and 2.0 μg/ml hygromycin B plus 0.4 M KCl. WT, wild-type; ΔΔ, *cta3*Δ *cfr1*Δ. **(C)** WT and *cfr1Δ* cells bearing an empty vector or with an integrative plasmid that allows *cta3*^+^ expression from the *nmt1*^+^ promoter were spotted on minimal medium without leucine (MM -leu) supplemented with KNO_3_ and incubated at 32°C for 5 days. **(D)** Cell extracts from the WT and *bch1Δ* strains bearing Cta3-GFP, incubated in YES before and after adding 0.6 M KCl, were subjected to SDS-PAGE, and were immunoblotted with monoclonal anti-GFP (upper panel) and anti-tubulin (lower panel; loading control) antibodies. The minutes indicate the time of incubation with 0.6 M KCl. The relative position of molecular weight markers is indicated on the right (KDa). The intensity of each Cta3-GFP band was relativized to the value for the corresponding tubulin band, and the values for the mutant were relativized to the value for the WT grown in KCl for the same time. **(E)** Micrographs of WT and *bch1*Δ cells bearing Cta3-GFP after 1 h of incubation in 0.6 M KCl. **(F)** Upper panel, the same experimental conditions as in **(E)**. The micrographs show cells with different Cta3 distribution in the plasma membrane. The asterisks denote regions with asymmetrical Cta3 distribution, and the arrowhead denotes a cell pole with subapical Cta3 distribution. Lower left panel, three-dimensional reconstructions of confocal images of the Cta3-GFP distribution in WT and *bch1Δ* cells incubated in KCl for 1 h. For the WT, cells with symmetric Cta3 distribution in the cell poles (a) and midzone (b and c) are shown. For *bch1*Δ, cells with abnormal distribution at the cell poles (a and c) and midzone (b and d) are shown. Right panel, the graph represents the percentage of WT and *bch1Δ* cells with different patterns of Cta3 distribution. The cartoon in the middle represents the different patterns that were scored. **(G)** The same experimental details as in **(E)**, but cells were incubated in KCl for 3 h. **(H)** Dot-plot representation of the fluorescence intensity of intracellular Cta3-GFP dots in WT and *bch1Δ* cells captured in three independent experiments. a. u., arbitrary units. **(I)** Quantification of the colocalization of Cta3-GFP and the TGN marker mCherry-FAPP (left panel) or the PVE marker mCherry-FYVE (right panel) in the WT and *bch1Δ* strains. The images in **(E–G)**, were acquired with a DeltaVision system and are SUM projections. Bar, 10 μm. In **(F,H,I)**, the cells were incubated in 0.6 M KCl for 1 h. For each value, the mean of three independent experiments, standard deviation, and statistical significance of the difference, are shown (*p < 0.05; **p < 0.01; ***p < 0.001; ****p < 0.0001). The statistical differences were determined using Tukey’s multiple comparison test **(D)**, Sidak’s multiple comparison test **(F,I)** and the *t*-test **(H)**.

*cfr1Δ* accumulated intracellular K^+^ and we could not identify a clear defect in an intake transporter/channel, which prompted us to investigate a potential defect in K^+^ extrusion. The *tup11*^+^ and *tup12*^+^ repressors regulate the expression of the Cta3 efflux ATPase in response to ionic but not osmotic stress (Nishikawa et al., 1999; Benito et al., 2002; Greenall et al., 2002). In agreement with this, we compared the growth of the exomer mutants on KCl and KNO_3_ with that of *cta3Δ* and the double mutants in *tup11^+^ tup12^+^* and *tup11Δ tup12Δ* backgrounds (Figure 4A). We found that the simultaneous deletion of exomer components and *cta3*^+^ led to sensitivity to low concentrations of KCl and KNO_3_, both in the presence and absence of the repressors, while *cta3Δ* was only sensitive to 0.6 M KCl in the *tup11Δ tup12Δ* background. Since Cta3 was originally described as an ATP-dependent Ca^2+^ pump (Ghislain et al., 1990; Halachmi et al., 1992), we also analyzed growth on CaCl_2_ plates and found that *cta3Δ* was only sensitive to high calcium concentrations, and only in the absence of the Tup regulators. Under these conditions, *cta3Δ bch1Δ* was sensitive to lower calcium concentrations. Altogether, these results showed genetic interaction between exomer and Cta3 for a function that seemed more specific for growth under potassium than under calcium stress. Regarding growth in the presence of hygromycin B, *cta3*Δ was not sensitive, and *cta3*Δ *cfr1*Δ (denoted by ΔΔ in the Figure 4B) was as sensitive as *cfr1*Δ. The addition of 0.4 M KCl improved *cfr1*Δ growth in the presence of the antibiotic. Interestingly, *cta3*Δ *cfr1*Δ did not grow in either 0.4 M KCl or 0.4 M KCl plus hygromycin B. These data suggested that the extrusion pump was required for the improvement of growth in the presence of the salt and the antibiotic and/or that in the absence of exomer and the extrusion pump there was strong K^+^ accumulation, which was deleterious for cells. We reasoned that if strong K^+^ sensitivity in the double mutants was the consequence of K^+^ hyperaccumulation, *cta3*^+^ overexpression should have the opposite effect. To test this hypothesis, we overexpressed *cta3*^+^ in the WT and *cfr1Δ* strains and assessed growth on KNO_3_ plates. The result was that overexpression of the extrusion pump alleviated the growth of *cfr1Δ* in the presence of high K^+^ concentrations (Figure 4C). These results showed a functional relationship between exomer and Cta3 that was related to defective K^+^ extrusion in the mutant.

Next, we aimed to understand the relationship between exomer and Cta3. We used western blotting to determine whether *cta3*^+^ was properly regulated in response to potassium in exomer mutants. As shown in Figure 4D, in both WT and *bch1*Δ strains Cta3-GFP was detected after 60 min of incubation in the presence of 0.6 M KCl, indicating timely expression, although the amount of the protein was higher in the mutant. Regarding protein localization, microscopy observation showed that 1 h after the addition of KCl most Cta3-GFP accumulated at the cell periphery of the sites of polarized growth (cell poles and equator), and that the intensity of the fluorescence in this location was similar in WT and mutant cells (Figure 4E and Supplementary Figure 2). Nevertheless, detailed observation showed that Cta3 distribution at the cell surface was different in both strains (Figure 4F). In the WT, the protein was observed symmetrically at the cell tips and neighboring areas, as well as at the cell midzone (upper and left panels in Figure 4F). In *bch1Δ*, there were cells whose fluorescent signal at both sides of the cell poles and/or midzone had different length (a distribution termed asymmetrical), and cells with fluorescence at the cell poles but not at the pole tips (subapical distribution). Quantification of the percentage of cells with different Cta3 distribution patterns demonstrated that in the WT about 10% of the cells exhibited asymmetrical and subapical Cta3 distribution, while this percentage was over 50% in the mutant (lower right panel in Figure 4F). Interestingly, the asymmetrical and subapical distribution was not observed after 3 h in KCl (Figure 4G). This result suggested that after arrival at the cell surface, Cta3 diffused along the PM.

Additionally, part of the protein resided in intracellular fluorescent dots (Figure 4E). Measurement of fluorescence intensity showed that the mutant had dots with significantly stronger fluorescence than that of dots in the WT (Figure 4H), indicating a larger amount of intracellular Cta3. Colocalization analyses with the TGN marker mCherry-FAPP and the PVE marker mCherry-FYVE demonstrated that in the WT most intracellular Cta3 dots corresponded to TGN, while only 2% of the dots corresponded to the PVE. In the absence of exomer, there was a change in Cta3 distribution, with a significant reduction in the fraction of Cta3 that populated the TGN and a significant increase in the fraction that populated the PVE (Figure 4I and Supplementary Figure 2).

In summary, these results demonstrated that exomer was required for efficient Cta3 transport from the TGN to the PM. The altered intracellular distribution might be related to abnormal Cta3 trafficking and/or to altered endosomal organization in the mutant. Moreover, although the absence of this complex did not block transport to the PM, it resulted in an abnormal distribution at the cell surface, which might contribute to K^+^ sensitivity.

### Exomer Mutants Exhibit Defects in Ca^2+^ Homeostasis and in Pkd2 Distribution

**FIGURE 5:**
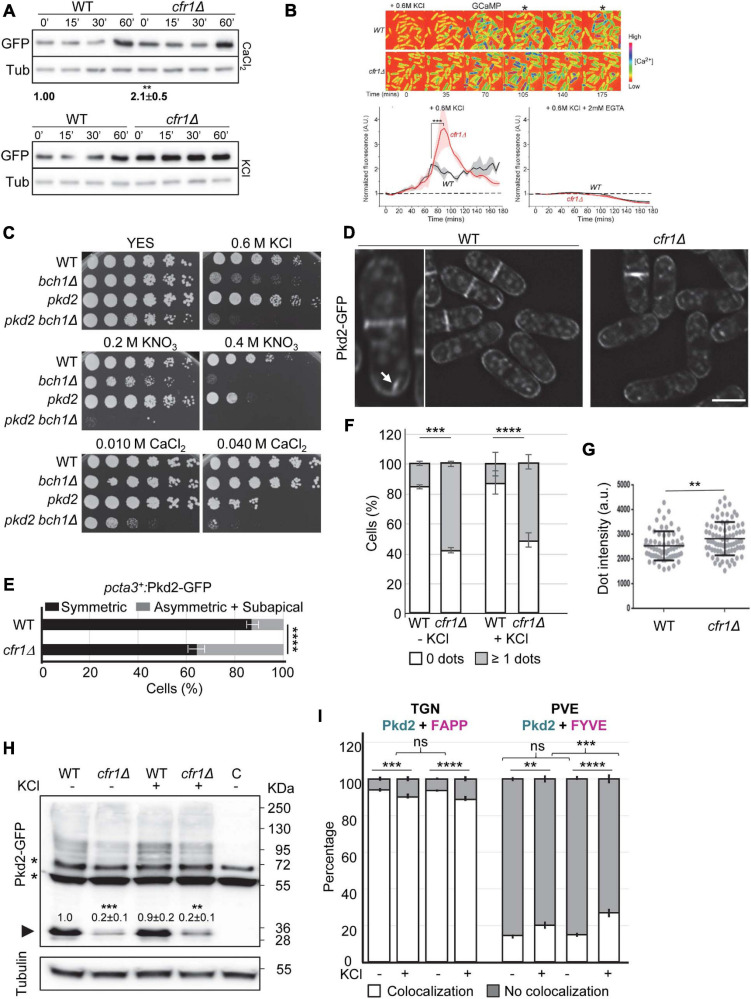
Relationship between exomer and calcium homeostasis. **(A)** Upper panel, cell extracts from the wild-type (WT) and *cfr1*Δ cells containing pCDRE-GFP and treated with 0.15 M CaCl_2_ for the indicated times (minutes). The numbers below the blot indicate the relative intensity of GFP/tubulin at 0′. Three independent experiments were performed; in all cases, the values for the mutant were relativized to the values for the WT, which were considered 1.00. The media, standard deviation and significance of the statistical differences for *cfr1*Δ are indicated. Lower panel, the same experimental details as in **(A)**, but the cells were treated with 0.6 M KCl for the indicated times. **(B)** Upper panel, time-lapse micrographs of wild-type (WT) and *cfr1Δ* cells after the addition of YES plus 0.6 M KCl. Numbers represent time points (minutes). The cells express the fluorescent GCaMP6s calcium indicator, and the colored spectrum shown on the right indicates the fluorescence level ([Ca^2+^]). The asterisks denote time-points when the calcium level in *cfr1Δ* was apparently different from that in the WT. Lower left panel, average time courses of the normalized GCaMP6s fluorescence intensity after the addition of the salt. Cloud represents the standard deviations. Data represent the average from two biological repeats. Lower right panel, the same experimental procedure, but the experiment was performed in the presence of KCl and EGTA. **(C)** Cells from the indicated strains were spotted on YES (rich medium) and YES supplemented with the indicated compounds. WT, wild-type; *pkd2*, *pkd2-81KD* mutant. **(D)** Micrographs of WT and *cfr1Δ* cells bearing Pkd2-GFP and incubated in YES. The panel on the left is an enlargement of a WT cell. The arrow denotes an intracellular Pkd2-GFP dot. Scale bar, 10 μm. **(E)** Quantitative analysis of the patterns of distribution of Pkd2-GFP in WT and *cfr1Δ* cells where *pkd2*^+^ was under the control of the *cta3*^+^ promoter. Cells were incubated in 0.6 M KCl for 1 h. **(F)** Percentage of WT and *cfr1Δ* cells, incubated in YES and YES with 0.6 M KCl for 1 h, that exhibited no fluorescent intracellular Pkd2-GFP dots, or at least one dot. **(G)** Dot-plot representation of the fluorescence intensity of intracellular Pkd2-GFP dots in WT and *cfr1Δ* cells incubated in 0.6 M KCl for 1 h. **(H)** Cell extracts from the indicated strains containing Pkd2-GFP incubated in YES with and without 0.6 M KCl. The lane on the right **(C)** corresponds to extracts from a strain without GFP, used to determine the unspecific bands, which are denoted by asterisks. The arrowhead denotes clipped GFP. Tubulin was used as a loading control. The intensity of each GFP band was relativized to the value for the corresponding tubulin band, and all the values were relativized to the value for the WT grown in YES. **(I)** Quantification of the colocalization of Pkd2-GFP and the TGN marker mCherry-FAPP (left panel) or the PVE marker mCherry-FYVE (right panel) in the WT and *cfr1Δ* strains incubated in 0.6M KCl for 1 h. In **(A,H)**, equal amounts of protein in the cell extracts were subjected to SDS-PAGE, and were immunoblotted with anti-GFP (upper panel) and anti-tubulin (lower panel; Tub, loading control) antibodies. In **(F–I)**, the cells were incubated in YES (KCl–) and in YES with 0.6M KCl (KCl+) for 1 h. For each value, the mean of three independent experiments, standard deviation, and statistical significance of the difference are shown. The statistical differences were determined using the *t*-test **(A,B,G)**, Sidak’s multiple comparisons test **(E,F)** and Tukey’s multiple comparisons test **(H,I)**. ns, non-significant; **p < 0.01; ***p < 0.001; ****p < 0.0001.

Since *cta3*Δ is defective in Ca^2+^ homeostasis (Ghislain et al., 1990; Halachmi et al., 1992) and exomer mutants exhibited mild sensitivity to CaCl_2_ (Figure 3D), we investigated the relationship between their sensitivity to KCl and Ca^2+^ homeostasis. Initially, we used calcineurin activation as a proxy to estimate the level of intracellular calcium. Quantitative western blotting was used to determine the amount of soluble GFP expressed from a calcineurin-responsive promoter (CDRE-GFP; Deng et al., 2006; Kume et al., 2011; Ma et al., 2011; Diessl et al., 2020). As expected, the amount of GFP increased when we added CaCl_2_ to the medium (upper panel in Figure 5A). A close observation of the results indicated that under basal conditions (0′), the amount of GFP was significantly greater in *cfr1*Δ than in the WT, which suggested increased intracellular Ca^2+^. Next, we added KCl to the cultures, and observed an increase for GFP in the WT but not in *cfr1*Δ (60′; lower panel in Figure 5A).

It was possible that the strong GFP signal detected in the mutant under basal conditions did not allow observing an increase. In addition, calcineurin response to Ca^2+^ and Cl^–^ are differentially regulated (Hirayama et al., 2003). To overcome these caveats, we analyzed calcium homeostasis after KCl treatment using a more direct and sensitive approach. We performed Ca^2+^ imaging using the fluorescent calcium indicator GCaMP and time-lapse microscopy (Poddar et al., 2021). Both WT and *cfr1Δ* cells were imaged after adding 0.6 M KCl to examine how cells responded to this stress. We found that the salt induced a similar gradual increase in the calcium level in both the WT and mutant. The Ca^2+^ level of the WT cells peaked after 75 min, reaching a two-fold increase relative to the baseline level (Figure 5B). However, in *cfr1Δ* cells the amount of Ca^2+^ continued increasing for another 15 min, and reached a peak that was more than three-fold the value of the baseline (Figure 5B). The maximum Ca^2+^ level in *cfr1Δ* was 67% higher than in the WT. After reaching the peak, the Ca^2+^ level stabilized in the WT, while it declined gradually in the mutant over the remaining time, and reached a level that was lower than that of the WT. These results showed that both the initial response and the adaptation to the presence of KCl were altered in the absence of exomer. To understand whether the cytoplasmic calcium increase was produced by influx from the exterior or by movements from internal reservoirs, we analyzed the Ca^2+^ level in the presence of EGTA. We found that the presence of the chelator in the medium completely blocked the increase in Ca^2+^ levels in both the WT and the mutant cells (Figure 5B). In summary, we concluded that Cfr1 modulated the cellular Ca^2+^ response to KCl stress by regulating some aspect of Ca^2+^ influx.

Therefore, we analyzed the relationship between exomer and proteins involved in calcium transport. First, we were interested in Pkd2, the *S. pombe* ortholog to the mammalian Transit Receptor Potential (TRP)-like polycystic-kidney-disease (PC2) ion channel (Palmer et al., 2005). TRP channels conduct Ca^2+^, Na^+^, and K^+^ ions (Dong et al., 2010). *pkd2Δ* is inviable and the *pkd2-81KD* mutant, where *pkd2*^+^ is under the control of the low-expression version of the *nmt1*^+^ promoter, is sensitive to 1 M KCl and CaCl_2_ (Palmer et al., 2005; Morris et al., 2019). To understand whether Pkd2 was an exomer cargo whose blockade in the TGN would lead to *cfr1Δ* and *bch1Δ* phenotypes, we compared the growth of *bch1Δ*, *pkd2-81KD* and the double mutant in the presence of K^+^ and Ca^2+^. *pkd2-81KD* (denoted by *pkd2* in the Figure 5C) grew better than *bch1Δ* on plates with potassium (0.6 M KCl and 0.4 M KNO_3_) and worse than *bch1Δ* on plates with 40 mM CaCl_2_. In all cases, the *pkd2-81KD bch1Δ* strain (denoted by *pkd2 bch1Δ* in the figure) was the most sensitive.

Although these results indicated that the phenotypes of exomer mutants were not due to Pkd2 blockade in the TGN, we analyzed Pkd2 distribution in the WT and mutants. We found that Pkd2-GFP was at the cell surface, with strong accumulation at the septal area in both strains (Figure 5D). Quantitative analyses did not detect significant differences between the strains regarding the distribution and intensity of Pkd2-GFP fluorescence at the cell surface, neither under basal conditions nor in KCl (Supplementary Figure 3). To understand whether Pkd2 distribution was abnormal a short time after induction, as we observed for Cta3, we expressed *pkd2*^+^ from the *cta3*^+^ promoter. Under these conditions, the percentage of cells with asymmetrical and subapical fluorescence distribution was significantly higher in *cfr1Δ* than in the WT (Figure 5E).

In addition, there was intracellular Pkd2-GFP fluorescence. All cells exhibited a faint signal that corresponded to the vacuoles, which in *S. pombe* are small and numerous, and some cells exhibited at least one bright intracellular dot (denoted by an arrow in Figure 5D). In the WT, less than 20% of the cells exhibited bright intracellular dots, while this number was over 40% in *cfr1Δ* (Figure 5F). This difference was evident in the absence of KCl, showing that it was produced by a lack of exomer. Moreover, the dots were brighter in the mutant than in the control (Figure 5G). Western blotting showed that stronger fluorescence was not due to higher levels of the protein (Figure 5H). Conversely, quantitation of the amount of clipped GFP, which normally serves as a proxy to estimate the abundance of transmembrane proteins, showed that there was less Pkd2-GFP in *cfr1Δ* than in the WT. Quantitative colocalization analyses showed that most of these dots corresponded to the TGN and that fewer dots corresponded to the PVE (Figure 5I and Supplementary Figure 3). Exposure to KCl resulted in a small but significant change in Pkd2 distribution, with a reduction in the number of dots that corresponded to the TGN and an increase in the number of dots that corresponded to the PVE. Pkd2 accumulation in the PVE in the presence of KCl was stronger in *cfr1Δ* than in the WT. Thus, the effect of potassium in Pkd2 intracellular distribution was enhanced by exomer deletion.

To gain more information about the relationship between exomer and calcium homeostasis, we analyzed its relationship with other proteins involved in the transport of this cation. Trp663 and Trp1322 are TRP-like channels (Ma et al., 2011), and therefore, we analyzed their sensitivity to potassium and their functional relationship with exomer. As shown in Supplementary Figure 4, their deletion neither produced sensitivity to KCl nor enhanced the sensitivity of *bch1Δ*. Conversely, double mutants were more sensitive to CaCl_2_ than single mutants. We also analyzed the relationship between exomer and the calcium channels Cch1 and Yam8 (Ma et al., 2011). *cch1Δ* was sensitive to low KCl concentrations, and both *cch1Δ cfr1Δ* and *yam8Δ cfr1Δ* were more sensitive than any of the single mutants. Interestingly, deleting *cch1*^+^ suppressed *cfr1Δ* sensitivity to CaCl_2_ (Supplementary Figure 4). Finally, we analyzed exomer relationship with Pmr1 and Pmc1, which are P-Type ATPases that participate in calcium transport and localize in the PM and the vacuole, respectively (Cortes et al., 2004). *pmr1Δ* was more sensitive to KCl than *cfr1Δ*, *pmc1Δ* was less sensitive than *cfr1Δ*, and the double mutants were the most sensitive (Supplementary Figure 4). Thus, there was a functional relationship between exomer and calcium transporters regarding growth in the presence of potassium. Regarding calcium, *pmc1Δ* was partially sensitive to a low CaCl_2_ concentration at which *pmc1Δ cfr1Δ* was unable to grow, and *pmr1Δ* was sensitive to 100 mM CaCl_2_ while *pmr1Δ cfr1Δ* was not, which suggested that an exomer-mediated process was deleterious for *pmr1Δ* in the presence of calcium.

Taken together, these experiments showed that calcium homeostasis was altered in exomer mutants, that small defects in the transport of Pkd2 might contribute to this alteration, and that Cch1 facilitates a calcium import that is deleterious for exomer mutants. Additionally, they suggested that exomer and the mechanisms that govern calcium homeostasis act in parallel to allow cell growth under K^+^ stress.

### The Prevacuolar Endosome Is Aberrant in Exomer Mutants Treated With KCl

**FIGURE 6:**
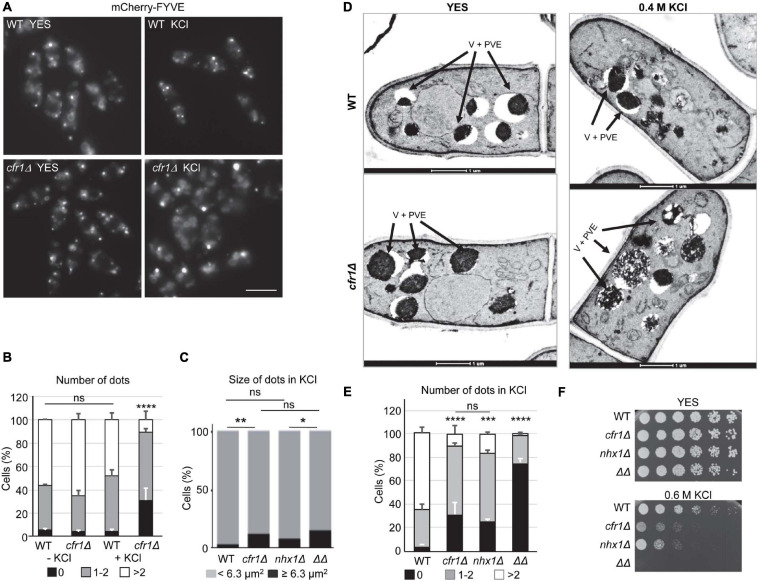
Analysis of the prevacuolar endosome in exomer mutants. **(A)** Wild-type (WT) and *cfr1Δ* cells with the PVE marker mCherry-FYVE incubated in YES and YES with 0.6 M KCl. Images are single planes captured with a Nikon Eclipse 90i. Bar, 10 μm. **(B)** The same experimental details as in **(A)**. The percentage of cells without mCherry-FYVE dots, 1-2 dots, and more than two dots were scored from the photographs. **(C)** The same experimental details as in **(A)**. The percentage of WT, *cfr1Δ*, *nhx1Δ*, and *cfr1Δ nhx1Δ* (ΔΔ) cells with mCherry-FYVE dots of the indicated size were scored. **(D)** Transmission electron microscopy of WT and *cfr1Δ* cells incubated with 0.4 M KCl for 1 h. Arrows denote vacuoles and prevacuolar endosomes (V + PVE). **(E)** The same experimental details as in **(A)**. The percentage of cells from the indicated strains without mCherry-FYVE dots, 1–2 dots, and more than two dots were scored. **(F)** Cells from the WT, *cfr1Δ*, *nhx1Δ*, and *cfr1Δ nhx1Δ* (ΔΔ) strains were spotted on YES and YES with 0.6 M KCl. In **(B,C,E)**, the media, standard deviation, and statistical significance of the differences from three independent experiments—determined using Tukey’s multiple comparisons test—are shown for each value (ns, non-significant; *p < 0.05; **p < 0.01; ***p < 0.001; ****p < 0.0001).

When we had performed colocalization analyses (Figures 4I, 5I), we had observed that the appearance of the mCherry-FYVE fluorescent dots in the *cfr1Δ* strain treated with KCl was different from that in the untreated cells (representative images are shown in the Figure 6A). Quantitative analysis showed that the percentage of cells with less than two dots was significantly higher in the mutant strain treated with KCl than in the untreated cells, and in the WT with and without KCl (Figure 6B). This increase was compensated with a reduced percentage of cells with more than two dots in the mutant. Additionally, the percentage of cells that exhibited large dots was significantly higher in *cfr1Δ* cells treated with KCl than in untreated cells (Figure 6C). To confirm the nature of the large aberrant dots in *cfr1*Δ cells exposed to KCl, we performed electron microscopy. *S. pombe* bears numerous small vacuoles scattered throughout the cytoplasm with empty areas and areas of electron-dense material, which might correspond to the multivesicular body (McCully and Robinow, 1971). The presence of 0.6 M KCl in the culture interfered with cell fixation; therefore, we incubated the cells with 0.4 M KCl for 1 h. Under these conditions, *cfr1Δ* cells exhibited very large vacuoles with dense content (Figure 6D), which supported the data obtained with mCherry-FYVE (Figures 6A,C).

Nhx1/Cpa1 is similar to *S. cerevisiae NHX1*, a Na^+^, K^+^/H^+^ antiporter that sequesters Na^+^ and K^+^ into the PVE/vacuoles, contributing to K^+^ tolerance, and controls multivesicular body-vacuole fusion, and *nhx1*Δ is sensitive to high K^+^ and hygromycin B (Gaxiola et al., 1999; Brett et al., 2005; Karim and Brett, 2018). Consequently, we analyzed the relationship between *S. pombe* Nhx1 and exomer. When we estimated the percentage of *nhx1Δ* and *cfr1Δ nhx1Δ* cells that exhibited large mCherry-FYVE dots in the presence of KCl, we found that the behavior of *nhx1Δ* was similar to that of the WT, and the behavior of *nhx1Δ cfr1Δ* (denoted by ΔΔ in the Figures 6C,E,F) was similar to that of *cfr1Δ* (Figure 6C). These data suggested that Nhx1 might not contribute to this phenotype. The number of mCherry-FYVE dots per cell was reduced in *nhx1*Δ, with a percentage of cells with more than two dots similar to that observed in *cfr1*Δ (Figure 6E). This reduction was greater in the *nhx1Δ cfr1Δ* double mutant. Similarly, we observed that the growth defect of both single mutants in 0.6 M KCl was similar, while *cfr1Δ nhx1Δ* could not grow at this concentration (Figure 6F). Finally, exomer mutants did not exhibit alterations in the distribution of Nhx1-GFP assessed by microscopy, or in its amount assessed by western blotting (Supplementary Figure 5).

Together, these results demonstrated that exposure to KCl altered the morphology/structure of the PVE in exomer mutants and strongly suggested that this alteration was unrelated to Nhx1.

### Lipid Distribution in the Plasma Membrane Is Altered in Exomer Mutants

**FIGURE 7:**
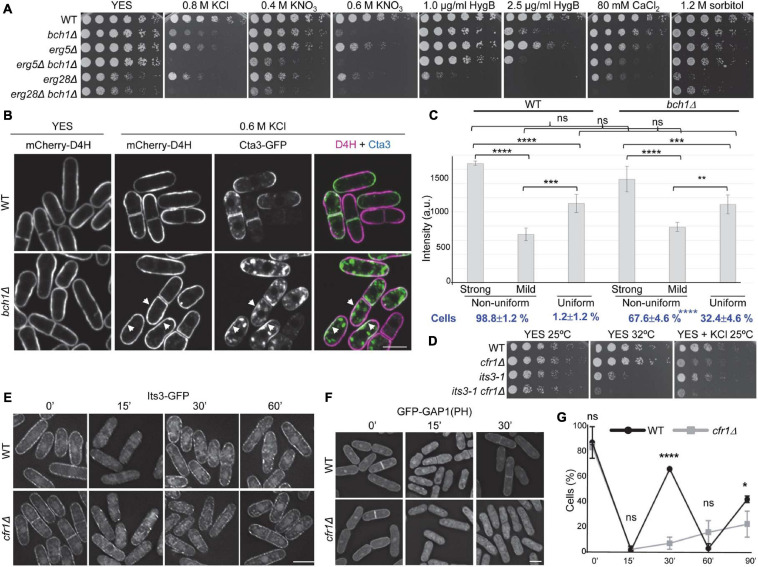
Relationship between exomer and plasma membrane lipids. **(A)** Cells from the indicated strains were spotted on YES (rich medium) and YES supplemented with the indicated compounds. **(B)** Wild-type (WT) and *bch1Δ* cells bearing the sterol-binding probe mCherry-D4H and Cta3-GFP incubated in YES and YES with 0.6 M KCl for 1 h were photographed under a confocal DragonFly microscope. Single-channel and merged images are SUM projections. Arrowheads denote membrane regions with strong GFP and mCherry signals. **(C)** Intensity of mCherry-D4H fluorescence (a.u., arbitrary units) in WT and *bch1*Δ cell regions with a uniform or non-uniform fluorescence distribution. In the cells with a non-uniform distribution, the intensity in the regions with strong and weak fluorescence was scored. The values from each of four independent experiments were relativized to the WT value in regions with strong fluorescence. The percentage of cells with a uniform and non-uniform fluorescence distribution is shown at the bottom in bold blue lettering. The numbers correspond to the mean, standard deviation, and statistical significance of the differences. The statistical differences were determined using Tukey’s (fluorescence intensity) and Sidak’s (percentage of cells) multiple comparisons tests. ns, non-significant; **p < 0.01; ***p < 0.001; ****p < 0.0001. **(D)** Cells from the indicated strains were spotted on YES (rich medium) and YES with 0.6 M KCl, and incubated at 25 and 32°C for 5 and 3 days, respectively. **(E)** WT and *cfr1Δ* cells bearing Its3-GFP incubated with 0.6 M KCl for the indicated times (minutes) were photographed with a Deltavision system. Images are SUM projections. **(F)** WT and *cfr1Δ* cells with the PI(4,5)P2-binding probe GFP-GAP1 incubated with 0.6 M KCl for the indicated times (minutes) were photographed with a Deltavision system. Images are SUM projections. **(G)** Percentage of WT and *cfr1Δ* cells with GFP-GAP1 fluorescence at the cell periphery. Cells were incubated with 0.6 M KCl and photographed at the indicated times (minutes). For each time, the mean of four independent experiments, standard deviation, and statistical significance of the differences, determined using Sidak’s multiple comparison test, are shown (ns, non-significant; *p < 0.05; ****p < 0.0001). In **(B,E,F)**, the scale bar corresponds to 10 μM.

The distribution of PM sterols influences the distribution of integral membrane proteins, including the ATPase Pma1, and has been related to membrane potential and salt tolerance (Iwaki et al., 2008; Kodedova and Sychrova, 2015). To gain information about the possible role of exomer in regulating sterol distribution, we first investigated the relationship between exomer, sterols, and stress by comparing the growth of exomer mutants with that of *erg5Δ* and *erg28Δ*, which are defective in sterol synthesis, and the corresponding double mutants. *erg5Δ* exhibited a sensitivity to K^+^ salts and hygromycin B, that was milder than that of exomer mutants, was slightly more sensitive to CaCl_2_, and grew well on sorbitol (Figure 7A). *erg5Δ bch1Δ* was more sensitive than the single mutants to all the compounds, except for sorbitol, to which none of the mutants was sensitive. Although *erg28Δ* exhibited slow growth on YES plates, it was possible to analyze its growth under stressing conditions. This mutant was sensitive to K^+^ salts, hygromycin B, CaCl_2_, and sorbitol (Figure 7A). *erg28Δ bch1Δ* was even more sensitive to all the stress sources except for sorbitol, a condition where it grew better than *erg28Δ*. These results suggested that exomer might have a function related to sterols but not related to the regulation of Erg5 and Erg28.

To gain more information about this function, we determined the distribution of sterols in the WT and *bch1Δ* strains using the mCherry-D4H sterol-binding probe. In YES, the distribution of this probe was similar in both strains (Figure 7B, YES). As previously described (Marek et al., 2020), the signal was present along the PM, although it was not uniform. Fluorescence was strong at the non-growing regions (the cell sides) and weak at the growing regions (the tips and cell equator before septation). After 1 h in 0.6 M KCl, we observed this mCherry-D4H distribution in 98.8% of the WT cells, while in the remaining 1.2% the probe distributed uniformly along the PM with intermediate fluorescence intensity (Figure 7B, 0.6 M KCl, and Figure 7C). In *bch1*Δ cells treated with KCl, the fluorescence intensity in the membrane regions with a strong signal was very variable, such that in some cells it was similar to that of the WT while in others it was close to that in cells with a uniform distribution. Because of this, the differences between the WT and *bch1Δ* values were not significant (Figures 7B,C). In addition to this variability, the most striking difference between the WT and mutant cells was the percentage of cells with uniform intermediate fluorescence, which increased from 1.2 to 32.4% in the mutant (Figure 7C). These results showed that the distribution of the sterol fraction accessible to the D4H probe was abnormal in exomer mutants exposed to KCl.

As described above, the Cta3 and Pkd2 distribution at the cell surface was abnormal in exomer mutants a short time after induction (Figures 4F, 5E). To understand whether there was some correlation between the distribution of Cta3 and sterols, we observed a strain bearing Cta3-GFP and mCherry-D4H under the microscope 1 h after adding 0.6 M KCl. Cta3 accumulated at the cell growing areas and was absent from the non-growing areas (Figures 4E,F, 7B). Thus, in the WT the membrane regions with intense green and red fluorescent signals did not overlap, which indicated that Cta3 populated membrane regions with low abundance of the sterol fraction accessible to D4H. In the mutant, Cta3-GFP exhibited asymmetric/subapical distribution at the cell poles/equator (Figures 4E,F, 7B). This distribution was different from that of sterols, which were not asymmetric. In consequence, a fraction of Cta3 populated membrane regions with a D4H fluorescence stronger than that in the regions where Cta3-GFP populated in the WT (some examples are denoted by arrows in Figure 7B).

The distribution of anionic lipids along cell membranes, together with their asymmetric distribution in both leaflets influences the charge and curvature of membranes, protein distribution, vesicle trafficking, signaling processes, and the positioning of the division plane (Gurtovenko and Vattulainen, 2008; Yeung et al., 2008; Platre and Jaillais, 2017; Caillaud, 2019). Although phosphoinositides (PIs) are not very abundant, they are highly anionic and therefore their presence and distribution have a significant impact in all these processes. Additionally, they accumulate selectively in different membranes, participating in local regulatory processes and contributing to the adequate directionality of vesicle trafficking (Behnia and Munro, 2005). In particular, PI(4,5)P2 is present in the PM, participates in secretion and endocytosis, and modulates the activity of K^+^ channels (Suh and Hille, 2005; Di Paolo and De Camilli, 2006). Consequently, we analyzed whether the absence of exomer led to alterations in this lipid. First, we compared the growth of *cfr1Δ* with that of *its3-1*, a thermosensitive mutant for the 1-phosphatidylinositol-4-phosphate 5-kinase Its3 (Zhang et al., 2000). We found that *cfr1Δ* enhanced *its3-1* thermosensitivity, and that *its3-1* enhanced *cfr1Δ* sensitivity to 0.6 M KCl, which showed genetic interaction (Figure 7D). Next, we determined the Its3-GFP distribution and found that, in the WT and *cfr1*Δ strains, this protein appeared as a string of fluorescent dots distributed along the PM (Figure 7E). Fifteen minutes after adding 0.6 M KCl, most of the fluorescent dots disappeared from the PM, and these dots were observed again in the PM 30 min after KCl addition in both strains. Interestingly, when we analyzed the behavior of a GFP-GAP1(PH) probe, which binds PI(4,5)P2 (Hammond et al., 2009), there were differences between the WT and *cfr1Δ* cells. In the WT, most of the fluorescence was observed uniformly along the PM before adding KCl (Figure 7F); additionally, there was weak fluorescence in the cytoplasm, which might be unspecific. Most of the fluorescence was cytoplasmic after 15 min in 0.6 M KCl and relocated to the PM after 30 min. The GFP-GAP1(PH) distribution in *cfr1Δ* was similar to that in the WT before KCl shock and after 15 min in KCl. However, the fluorescence did not relocate to the PM after 30 min (Figure 7F). Quantitative analysis of the behavior of GFP-GAP1(PH) for a long time unveiled a dynamic behavior in the WT, with cycles of association to and dissociation from the PM (Figure 7G). Since this dynamic behavior was not observed for the enzyme that synthesizes PI(4,5)P2, it was probably produced by changes in Its3 activity or in the accessibility of the probe to PI(4,5)P2. In the mutant, the probe dissociated from the PM after 15 min in KCl, and it did not associate with it completely even 90 min after KCl addition.

In summary, in the absence of exomer and in the presence of KCl, the distribution of sterols and PI(4,5)P2 was different from that of the WT, which might contribute to altered membrane polarization and ion homeostasis in the mutant.

### Phenotype of Mutants Defective in Protein Sorting in the TGN

**FIGURE 8:**
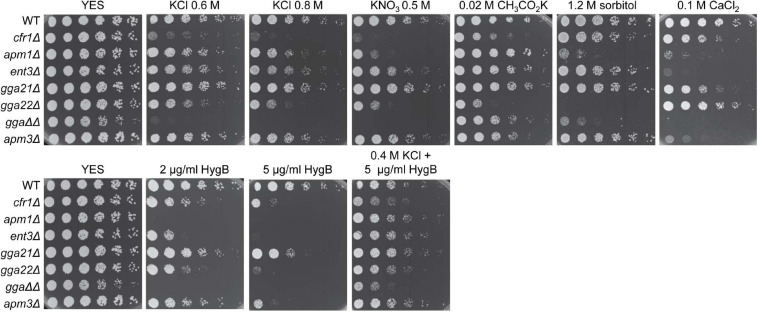
Sensitivity of mutants defective in clathrin adaptors to stress. Cells from the indicated strains were spotted on YES (rich medium) and YES supplemented with the indicated compounds, and incubated at 28°C for 4 days. WT, wild-type; *gga*ΔΔ, *gga21*Δ *gga22*Δ.

The phenotype observed in exomer mutants might not be specific to the absence of this complex but common to all mutants defective in protein sorting in the TGN. To investigate this possibility, we analyzed the growth of mutants deleted for clathrin adaptors that facilitate protein delivery to the PM (*apm1*Δ), PVE (*ent3*Δ, *gga21*Δ, *gga22*Δ, *gga21*Δ *gga22*Δ), and vacuole (*apm3*Δ) in the presence of K^+^ and Ca^2+^ salts, sorbitol, hygromycin B, and KCl plus hygromycin B. We found that *apm1*Δ, *gga22*Δ, and *gga21*Δ *gga22*Δ (denoted by *ggaΔΔ* in the Figure 8) were sensitive to both high concentrations of K^+^ salts and sorbitol (Figure 8), which showed that they were defective in growth under osmotic stress. In addition to *cfr1*Δ, the only mutant sensitive to potassium acetate was *gga22*Δ, which was the only mutant that grew on CaCl_2_ plates as efficiently as the WT. *ent3Δ* and *apm3Δ* were slightly sensitive to high concentrations of KCl and KNO_3_ and were very sensitive to CaCl_2_. All mutants exhibited a degree of sensitivity to hygromycin B that was alleviated by KCl, an indication of membrane hyperpolarization.

These results demonstrated that exomer mutants shared a defect in membrane polarization with other mutants defective in trafficking from the TGN but exhibited a unique combination of additional phenotypes related to alterations in K^+^ and Ca^2+^ homeostasis.

## Discussion

### K^+^ Sensitivity in Exomer Mutants

Potassium is the most abundant ion inside cells, and several yeast mutants are unable to grow when this ion is scarce (Calero et al., 2000; Calero and Ramos, 2003; Arino et al., 2014, 2019). Most of the mutants described as sensitive to high levels of K^+^ are also sensitive to sorbitol, demonstrating that they are unable to cope with osmotic rather than ionic stress. *S. pombe* exomer mutants were sensitive to different K^+^ salts at various concentrations but not to sorbitol, showing that exomer is necessary to sustain cell growth in the presence of potassium salts.

Exomer is a Golgi protein complex involved in the trafficking from the TGN to the plasma membrane of selected cargoes, which are blocked in the TGN in exomer mutants (Sanchatjate and Schekman, 2006; Trautwein et al., 2006; Wang et al., 2006; Paczkowski et al., 2012, 2015; Paczkowski and Fromme, 2014; Huranova et al., 2016). If a K^+^ influx transporter were an exomer cargo, the expected phenotype for exomer mutants would be a reduction in K^+^ uptake. The following results negated the hypothesis that the K^+^ sensitivity of exomer mutants was a consequence of the defect in the delivery of a particular K^+^ influx transporter/channel to the PM: (i) *cfr1*Δ accumulated more K^+^ than the WT, (ii) none of the mutants lacking transporters/channels exhibited the same phenotype as *cfr1*Δ, and (iii) in general, double mutants lacking exomer and these transporters/channels were more sensitive to K^+^ than the corresponding single mutants. Thus, although we could not disregard the hypothesis that an unknown transporter is an exomer cargo whose deletion promoted K^+^ uptake by low-affinity transporters (Bihler et al., 2002), it was possible that the phenotype of exomer mutants was produced by different and/or additional reasons. Since unspecific ion uptake happens in cells with membrane hyperpolarization (Madrid et al., 1998; Calero et al., 2000), hyperpolarization might contribute to K^+^ sensitivity. In fact, high ATPase activity and a sensitivity to hygromycin B that was alleviated by external K^+^ demonstrated membrane hyperpolarization in exomer mutants.

Potassium accumulation might be an additive effect of an increased unspecific uptake due to membrane hyperpolarization and reduced export. *S. pombe* exomer mutants accumulated more K^+^ and more Cta3—the K^+^-specific extrusion pump—than the WT and, interestingly, budding yeast exomer mutants accumulated less K^+^ and less Ena1—one of the extrusion pumps similar to Cta3—than the WT (Benito et al., 2002; Anton et al., 2017). Thus, in both organisms, the variations in the total level of intracellular K^+^ were the opposite to those expected according to the amount of the extrusion pump. Therefore, if a defect in these pumps was responsible for the altered intracellular K^+^ level, the reason was not the amount of protein. Our results showed mild defects in the trafficking of Cta3, which resulted in partial intracellular retention and mis-sorting to the PVE. Moreover, the fraction of the protein that reached the PM exhibited an abnormal distribution in the membrane regions of polarized growth. Notably, *S. cerevisiae* exomer mutants were also defective in the polarization of Ena1 (Anton et al., 2017). Thus, although the final output in terms of sensitivity to specific ions might be different because of the adaptation of each yeast to a different environment (Benito et al., 2002), in both organisms, exomer mutants were defective in the polarization of an extrusion pump, which might contribute to their abnormal K^+^ levels and to their sensitivity to alkali metal cations.

In different organisms, Ca^2+^ is important for the homeostasis of other ions, including K^+^ (Sarkadi et al., 1985; Flatman, 1987; Seifikalhor et al., 2019). The mild sensitivity to Ca^2+^ and the genetic interaction between mutants defective in Ca^2+^ transporters/channels/pumps and exomer mutants demonstrated a relationship between exomer and calcium. The fact that genetic interaction was positive or negative depending on the transporter suggested that this relationship was complex. Our results suggested that the level of intracellular Ca^2+^ was higher in exomer mutants than in the WT, and showed that elevating the level of extracellular K^+^ produced a Ca^2+^ spike, whose intensity was stronger in *cfr1Δ* than in the WT. These results showed altered calcium homeostasis under basal conditions and in response to high K^+^. In turn, since calcium is important for the regulation of K^+^ transporters (Casado et al., 2010; Capera et al., 2019), these alterations probably contributed to altered K^+^ homeostasis in exomer mutants. Additionally, the distribution of the calcium channel Pkd2 was abnormal inside the cells and at the PM, where it was asymmetrical at the sites of polarized growth a short time after induction. Since Pkd2 likely mediates the calcium level during cytokinesis (Morris et al., 2019), its abnormal distribution at the midzone might contribute to the generation of the abnormal septa observed in the mutants incubated in KCl (Hoya et al., 2017).

The presence of ion transporters, channels, and pumps in the PM depends on vesicle transport (Mulet et al., 2013; Capera et al., 2019). Additionally, these proteins distribute into membrane microdomains that provide the appropriate environment for their functionality (Martens et al., 2004; Dart, 2010; Capera et al., 2019). Since the presence of specific lipids defines these microdomains, the lipid composition of biological membranes determines the distribution and activity of ion transporters. Our results showed that the distribution of sterols and PI(4,5)P2 in the PM was altered in exomer mutants exposed to KCl. Consequently, under these conditions, Cta3, Pkd2, and probably other ion transporters, as well as the PM ATPase Pma1, populate membrane regions with altered lipid composition and/or distribution, which probably alters their activity. Their abnormal distribution and activity might produce local imbalances of ions and protons. These imbalances might alter vesicle generation and/or fusion in the PM, which would affect the lipid distribution that, in turn, would result in defective vesicle trafficking (Dong et al., 2010; Scott and Gruenberg, 2011; Martin, 2015). In fact, Pkd2 deletion alters protein trafficking (Aydar and Palmer, 2009). Local K^+^ and Ca^2+^ imbalances, and defects in traffic would contribute to K^+^ sensitivity in *S. pombe* exomer mutants.

In addition to their relevance in the PM, the presence of ion transporters in the membrane of intracellular organelles is important for the establishment and maintenance of ionic gradients along the endosomal system (Dong et al., 2010; Scott and Gruenberg, 2011). Vesicle trafficking ensures the localization of ion transporters in the appropriate organelle (Capera et al., 2019; Zimmermannova et al., 2019) and, as mentioned, the action of transporters influences vesicle trafficking. Therefore, it was possible that the mild defects in the endosomal system of exomer mutants (Hoya et al., 2017) contributed to abnormal intracellular ion homeostasis that, in turn, would alter endosomal integrity, vesicle trafficking, and lipid distribution in the PM. The presence of high external K^+^ amplified these defects (Figure 6), which would contribute to sensitivity to the ion. The pattern of stress sensitivities of mutants defective in clathrin adaptors, whose endosomes are altered (Kita et al., 2004; Hoya et al., 2017; Yanguas et al., 2019), demonstrated that their PM was hyperpolarized. This agreed with the role of the endosomal system in pH and ion homeostasis, and with results demonstrating a link between defects in the endosomal systems and sensitivity to hygromycin B (Banuelos et al., 2010; Scott and Gruenberg, 2011; Ejzykowicz et al., 2017). Nevertheless, the growth of mutants lacking exomer and clathrin-associated adaptors in the presence of various salts and sorbitol were different. Thus, the phenotype of exomer mutants was probably the result of the combination of different defects.

**FIGURE 9:**
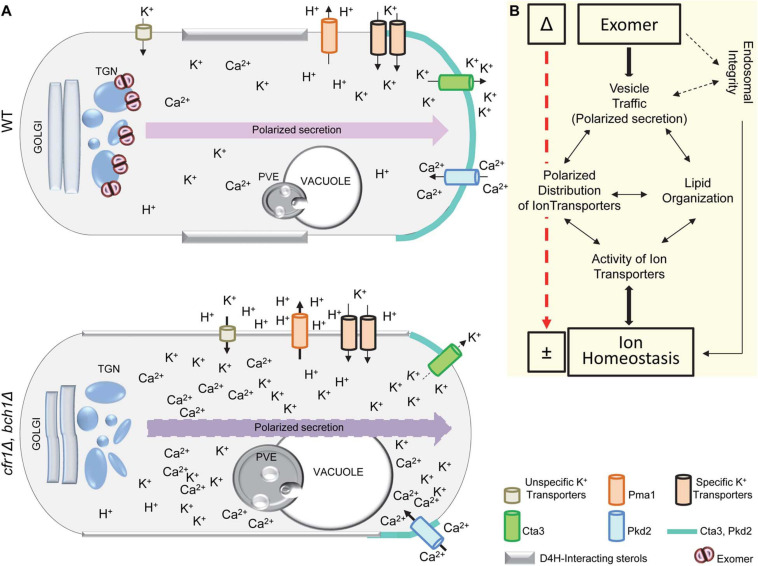
Schematic overview of the phenotypes observed in exomer mutants. **(A)** The cartoon in the upper panel (WT) represents a wild-type cell, and the cartoon in the lower panel (*cfr1*Δ, *bch1*Δ) represents an exomer mutant cell. In the mutant, there were alterations in the morphology of the Golgi, *trans*-Golgi network (TGN) (Hoya et al., 2017) and the prevacuolar endosome (PVE), in the polarized secretion and distribution of Cta3 and Pkd2, and in the distribution of some lipids. The ATPase Pma1 was hyperactive, resulting in plasma membrane (PM) hyperpolarization. PM hyperpolarization and abnormal Cta3 activity would result in enhanced K^+^ influx—probably through unspecific transporters—and reduced K^+^ efflux, leading to increased K^+^ accumulation. The abnormal distribution and/or activity of Pkd2—and maybe other Ca^2+^ channels—would result in increased calcium influx and accumulation. Thick arrows denote ion fluxes that would be enhanced in the mutant with respect to the WT, and the thin dashed arrow denotes fluxes that would be reduced. Some phenotypes were enhanced in the presence of KCl, and others were only observed under these conditions. **(B)** Interplay between the processes involved in the maintenance of ion homeostasis in wild-type cells. Exomer participates in vesicle traffic and facilitates polarized secretion that results in polarized distribution of ion transporters and in an adequate lipid organization in the PM. The polarized distribution of ion transporters and the adequate lipid organization modulate each other, guaranteeing adequate vesicle traffic, and resulting in proper activity of ion transporters. Additionally, exomer is required for the integrity and/or morphology of endosomes, either because of its regulation of vesicle transport or because of some unknown function (dashed arrows). Endosomal integrity is required for vesicle trafficking; therefore, it influences polarized secretion. Moreover, endosomes participate in the maintenance of intracellular ionic gradients and, therefore, their integrity is required for ion homeostasis. In exomer mutants (Δ), the coordination between these processes is altered (red dashed arrow), leading to abnormal ion homeostasis (±).

In summary, *S. pombe* exomer mutants exhibited a plethora of mild defects in several processes related to vesicle transport and ion homeostasis, as summarized in Figure 9A, and these defects were probably enhanced by the presence of K^+^ salts. The fact that the defects were mild and probably local explains why most of *cfr1*Δ cells recovered from stress when they were transferred from a medium with KCl to a medium with normal K^+^ levels (Figure 1B). Since many of these processes are interrelated (Figure 9B), we cannot ignore the possibility that all defects might be an indirect consequence of the alteration in a unique process. However, the genetic interaction between exomer mutants and the mutants that we analyzed indicated that the absence of exomer altered several processes. Moreover, our results with double and triple mutants indicated that, in several cases, the effects were additive. This notion was supported by the fact that *cfr1*^+^ was the only suppressor in a multicopy-suppressor screening designed to identify genes that overcame *cfr1*Δ sensitivity to KCl (our unpublished results). Therefore, it seems more plausible that several aspects of vesicle trafficking and ion homeostasis converge in exomer. Thus, exomer would be part of a hub where the mechanisms that regulate vesicle trafficking and ion homeostasis connect and are coordinated.

### Exomer and Polarized Secretion

Chs3, Fus1, and Pin2 are transmembrane proteins that localize in the sites of polarized growth. They are considered *bona fide* exomer cargoes because they interact with exomer components and remain in the TGN in the absence of this protein complex (Santos and Snyder, 1997, Santos and Snyder, 2003; Sanchatjate and Schekman, 2006; Trautwein et al., 2006; Barfield et al., 2009; Rockenbauch et al., 2012; Ritz et al., 2014). Skg6 is a polarized transmembrane protein captured in a pull-down screening designed to identify exomer cargoes (Ritz et al., 2014). Surprisingly, Skg6 localized to the plasma membrane in exomer mutants, a result that led to the conclusion that it was not a cargo (Ritz et al., 2014). Later, analyses of the sensitivity of exomer mutants to cations demonstrated that a significant percentage of cells did not exhibit Ena1 or the tryptophan transporter Tat2 in the PM of small buds (Anton et al., 2017, 2021). Moreover, an analysis of the regulation of chitin synthesis by exomer in other organisms, demonstrated that in *C. albicans* exomer mutants part of the chitin synthase CaChs3 was intracellular, and that the fraction of the enzyme that reached the PM was barely detected at the small buds and hyphal tips (Anton et al., 2018). Here, we demonstrated that *S. pombe* exomer mutants had defects in the polarized distribution of Cta3 and Pkd2. These results and the facts that (i) *S. pombe* bears the simplest functional exomer—whose ChAP is close to the ancient one (Martin-Garcia et al., 2011; Ramirez-Macias et al., 2018); (ii) Cta3 is close to the original K^+^-ATPase (Benito et al., 2002); (iii) exomer function as a dedicated cargo adaptor is a recent acquisition (Ramirez-Macias et al., 2018), strongly suggest that the original exomer function was related to polarized secretion. Notably, the subapical/asymmetrical distribution of Ena1, Cta3, and Pkd2 in the mutants was only evident a short time after induction. Therefore, it would be interesting to analyze the distribution of Skg6 and other potential cargoes under the same conditions. This would clarify whether the regulation of polarized secretion by exomer is frequent for transmembrane proteins that do not depend on it for exiting the TGN, or whether it is restricted to a small group of proteins that include several transporters.

After arrival at the cell surface, these proteins would move along the PM of the cell bud/pole/midzone by lateral diffusion (Valdez-Taubas and Pelham, 2003), explaining why their polarization defect would not be detected long after induction. Thus, it is possible that the proteins arrive at the PM in a similar way in the WT and mutants, and that the aberrant distribution of sterols, PI(4,5)P2 and maybe other anionic lipids as phosphatidylserine slows their lateral diffusion in the mutant, resulting in their transient defective localization. Nevertheless, there are other possibilities that must be considered and addressed in the future. Some possible and mutually non-exclusive functions of exomer in polarized secretion would be to facilitate (i) proper organization of lipid microdomains in the TGN, and/or the interaction between the proteins that promote vesicle budding and these microdomains, (ii) cargo oligomerization, (iii) the contact between the vesicle and myosin type V, and (iv) the establishment of the sites for polarized growth. Future studies should address these possibilities.

## Data Availability Statement

The original contributions presented in the study are included in the article/Supplementary Material, further inquiries can be directed to the corresponding author/s.

## Author Contributions

SM, EM-R, AP, JM, QC, and M-HV conducted the experiments and generated resources. SM, EM-R, AP, JM, PP, QC, and M-HV analyzed the results and provided the intellectual input. M-HV wrote the manuscript and obtained the financial support. All the authors have read, corrected, and approved the final manuscript.

## Conflict of Interest

The authors declare that the research was conducted in the absence of any commercial or financial relationships that could be construed as a potential conflict of interest.

## Footnotes

1 https://www.pombase.org/

## Supplementary Material

Supplementary Figure 1 Growth on KCl of mutants deleted for genes annotated as ion transporters or channels. **(A)** The indicated strains were spotted on YES and YES with 1 M KCl and incubated at 32°C for 3 days. **(B)** Predicted product of the genes deleted in the strains analyzed in **(A)**, as indicated in PomBase (https://www.pombase.org/).

Supplementary Figure 2 **(A)** Fluorescence intensity of Cta3-GFP in the cell surface of the wild-type (WT) and *bch1Δ* strains in the presence of 0.6 M KCl for 1 h. For each value, the mean of three independent experiments, standard deviation, and statistical significance of the difference, determined using the *t*-test are shown. ns, non-significant. **(B)** Colocalization between Cta3-GFP and the TGN marker mCherry-FAPP or the PVE marker mCherry-FYVE. Images are medial planes captured with a confocal spinning-disk microscope. Arrows denote dots where the GFP and mCherry signals colocalized. Bar, 10 μm.

Supplementary Figure 3 **(A)** Fluorescence intensity of Pkd2-GFP in the cell surface of the wild-type (WT) and *cfr1Δ* strains in the presence of 0.6 M KCl for 1 h. For each value, the mean of three independent experiments, standard deviation, and statistical significance of the difference, determined using the Tukey’s test are shown. ns, non-significant. **(B)** Colocalization between Pkd2-GFP and the TGN marker mCherry-FAPP or the PVE marker mCherry-FYVE. Cells from the wild-type (WT) and *cfr1Δ* strains grown in YES and YES with 0.6 M KCl for 1 h were photographed under a confocal spinning-disk microscope. Images are medial planes. Arrows denote dots where the GFP and mCherry signals colocalized. Bar, 10 μm.

Supplementary Figure 4 Analysis of the relationship between exomer and calcium transporters in the presence of the indicated concentrations of potassium and calcium chlorides. **(A)** Genetic interaction between *bch1Δ* and mutants for Transient Receptor Potential (TRP) Ca^2+^ channels. **(B)** Genetic interaction between *cfr1Δ* and mutants for the Ca^2+^ ion import channels Cch1 and Yam8. **(C)** Genetic interaction between *cfr1Δ* and mutants for the plasma membrane Ca^2+^ P-type ATPase Pmr1 and the vacuolar Ca^2+^ P-type ATPase Pmc1.

Supplementary Figure 5 **(A)** Cells from the wild-type (WT) and *cfr1Δ* strains bearing Nhx1-GFP and incubated in YES and YES with 0.6 M KCl for 1 h were photographed with a DeltaVision system. Images are SUM projections. Bar, 10 μm. **(B)** The same experimental conditions as in **(A)**, but the cells were broken and the cell extracts were subjected to SDS-PAGE and immunoblotted with anti-GFP (upper panel) and anti-tubulin (lower panel; loading control) antibodies. Extracts from a strain that did not express Nhx1-GFP were loaded in the first lane (C) to identify unspecific bands, which are denoted by asterisks. The intensity of each Nhx1-GFP band was relativized to the value for the corresponding tubulin band, and all the values were relativized to the value for the WT grown in YES. For each value, the mean of three independent experiments, standard deviation, and statistical significance of the difference, determined using the Tukey’s multiple comparisons test are shown (ns, non-significant).

Supplementary Table 1 List of strains used in this work.
