# Supplementary figures and images for: Exomer Is Part of a Hub Where Polarized Secretion and Ionic Stress Connect
Source: Front Microbiol. 2021 Jul 19;12:708354. doi: 10.3389/fmicb.2021.708354 (PMC8326576; doi:10.3389/fmicb.2021.708354)

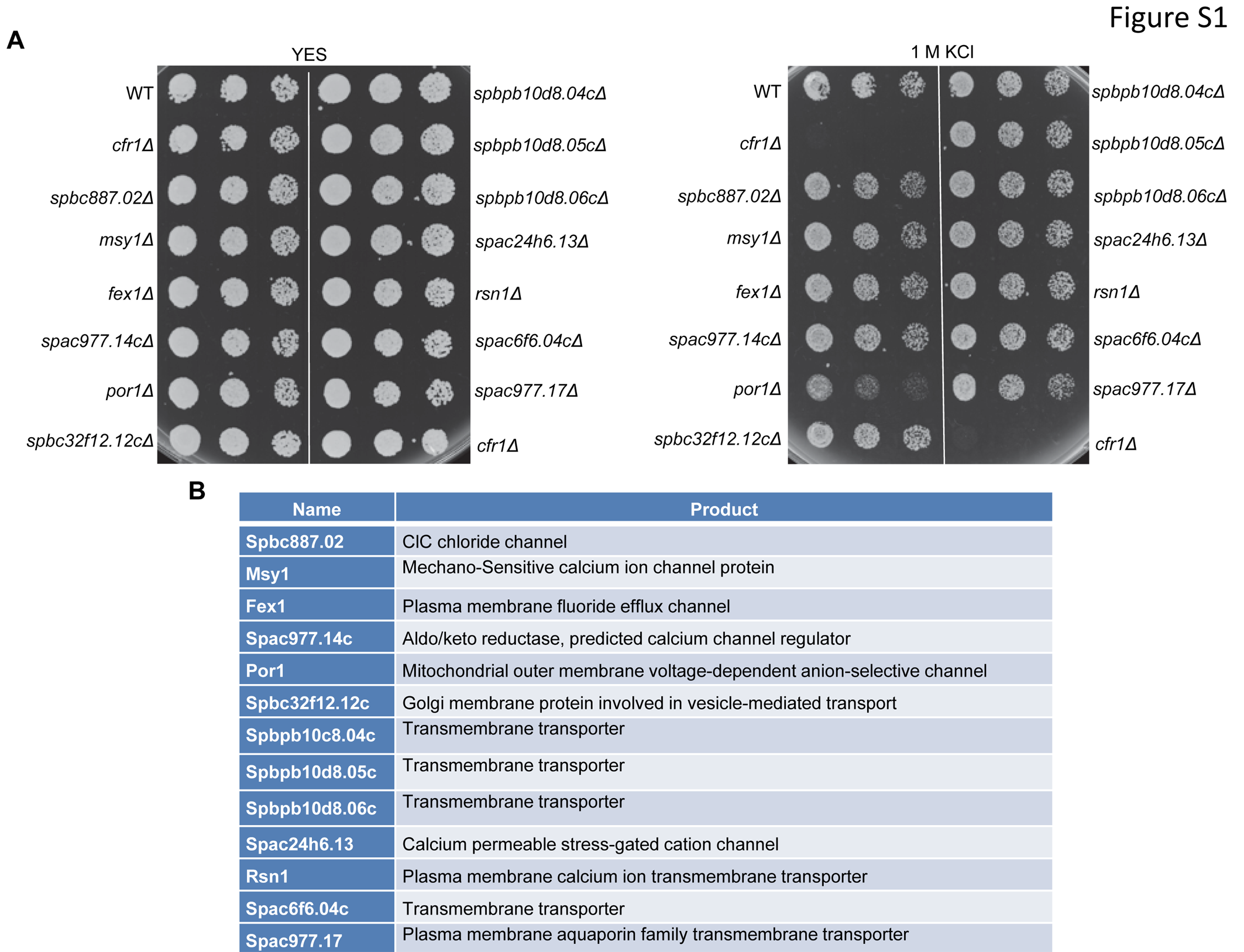

Supplement: Supplementary Figure 1 — Growth on KCl of mutants deleted for genes annotated as ion transporters or channels. (A) The indicated strains were spotted on YES and YES with 1 M KCl and incubated at 32°C for 3 days. (B) Predicted product of the genes deleted in the strains analyzed in (A), as indicated in PomBase (https://www.pombase.org/). [file Image_1.TIF]

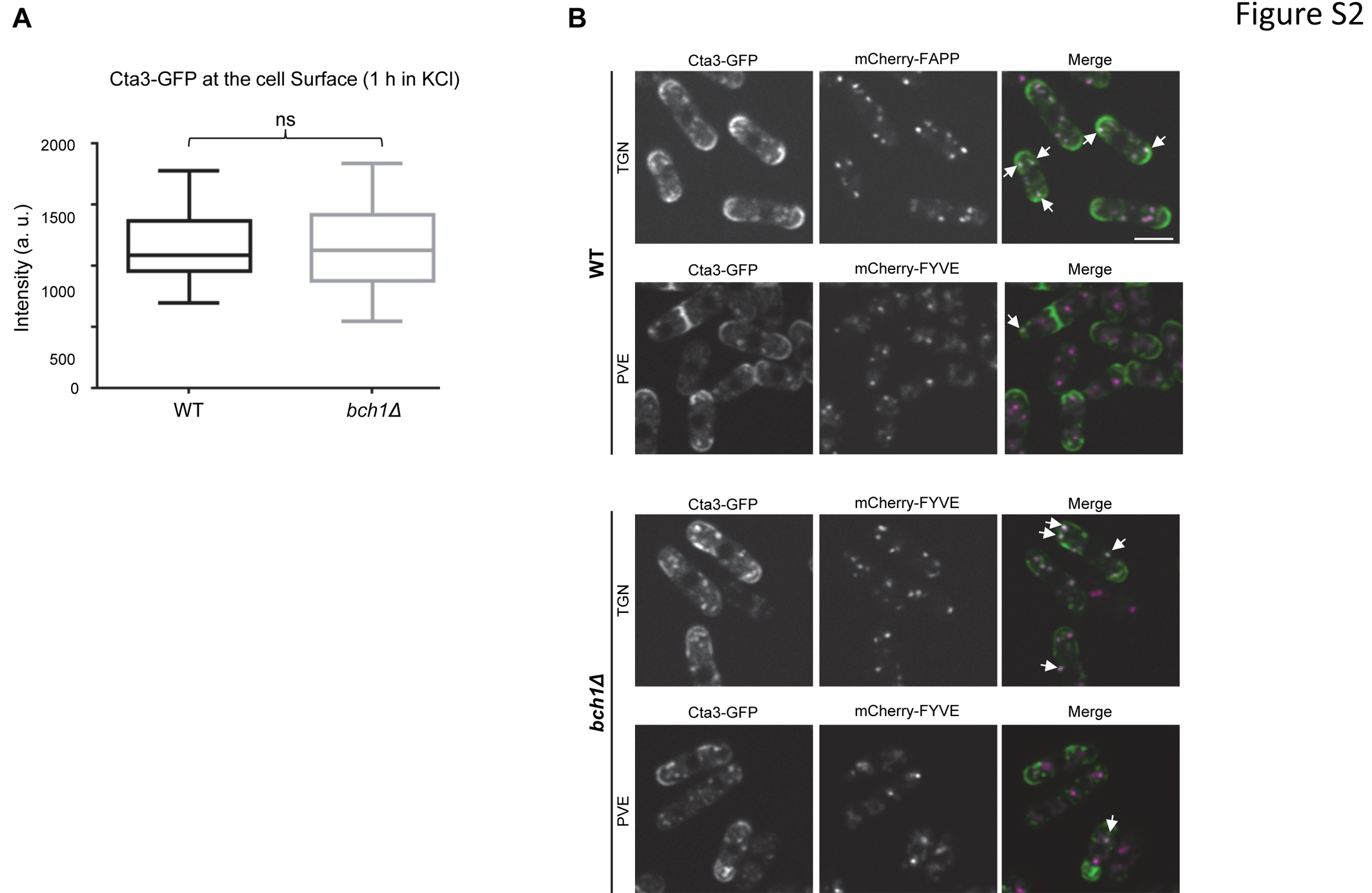

Supplement: Supplementary Figure 2 — (A) Fluorescence intensity of Cta3-GFP in the cell surface of the wild-type (WT) and bch1Δ strains in the presence of 0.6 M KCl for 1 h. For each value, the mean of three independent experiments, standard deviation, and statistical significance of the difference, determined using the t-test are shown. ns, non-significant. (B) Colocalization between Cta3-GFP and the TGN marker mCherry-FAPP or the PVE marker mCherry-FYVE. Images are medial planes captured with a confocal spinning-disk microscope. Arrows denote dots where the GFP and mCherry signals colocalized. Bar, 10 μm. [file Image_2.TIF]

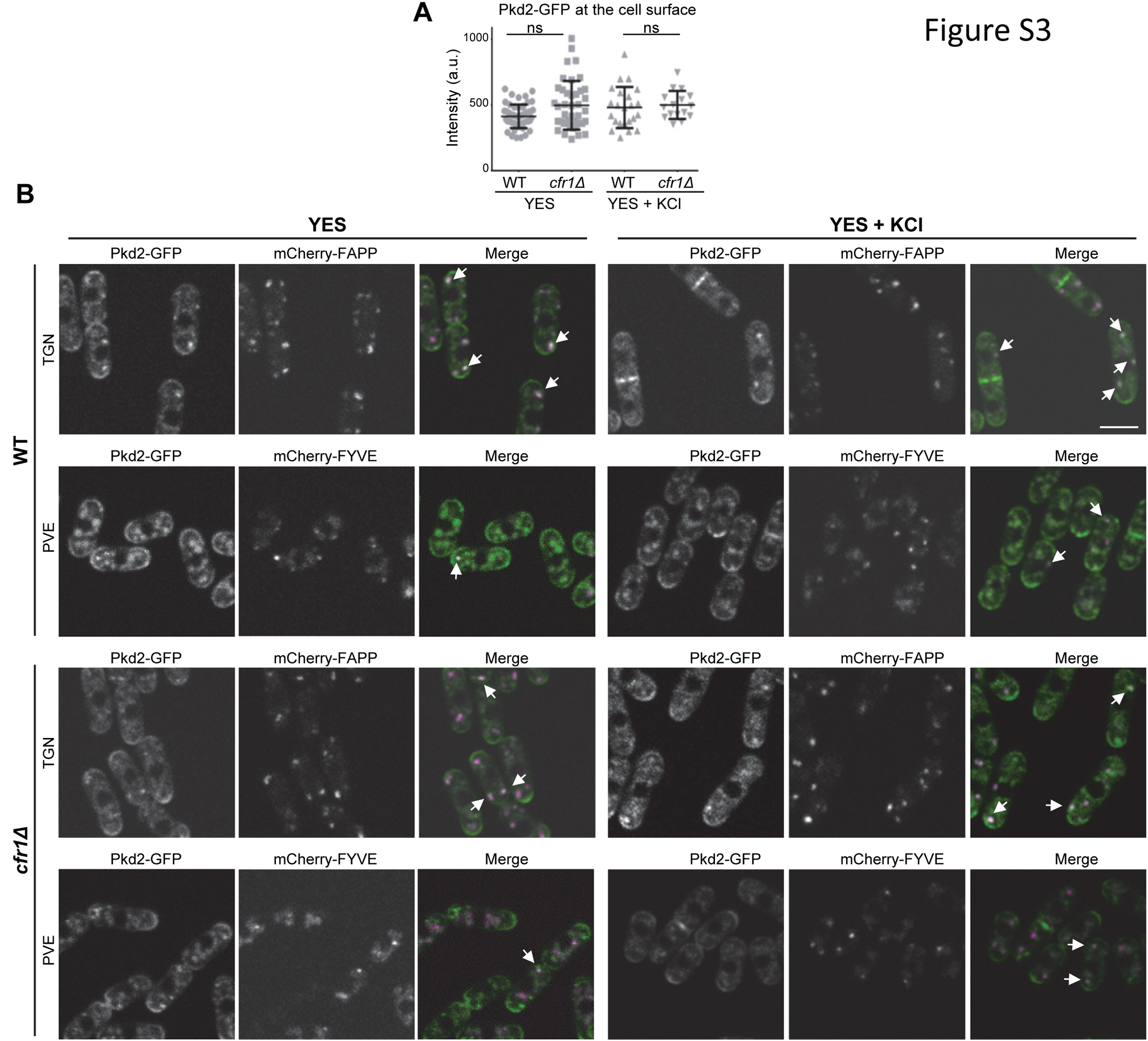

Supplement: Supplementary Figure 3 — (A) Fluorescence intensity of Pkd2-GFP in the cell surface of the wild-type (WT) and cfr1Δ strains in the presence of 0.6 M KCl for 1 h. For each value, the mean of three independent experiments, standard deviation, and statistical significance of the difference, determined using the Tukey’s test are shown. ns, non-significant. (B) Colocalization between Pkd2-GFP and the TGN marker mCherry-FAPP or the PVE marker mCherry-FYVE. Cells from the wild-type (WT) and cfr1Δ strains grown in YES and YES with 0.6 M KCl for 1 h were photographed under a confocal spinning-disk microscope. Images are medial planes. Arrows denote dots where the GFP and mCherry signals colocalized. Bar, 10 μm. [file Image_3.TIF]

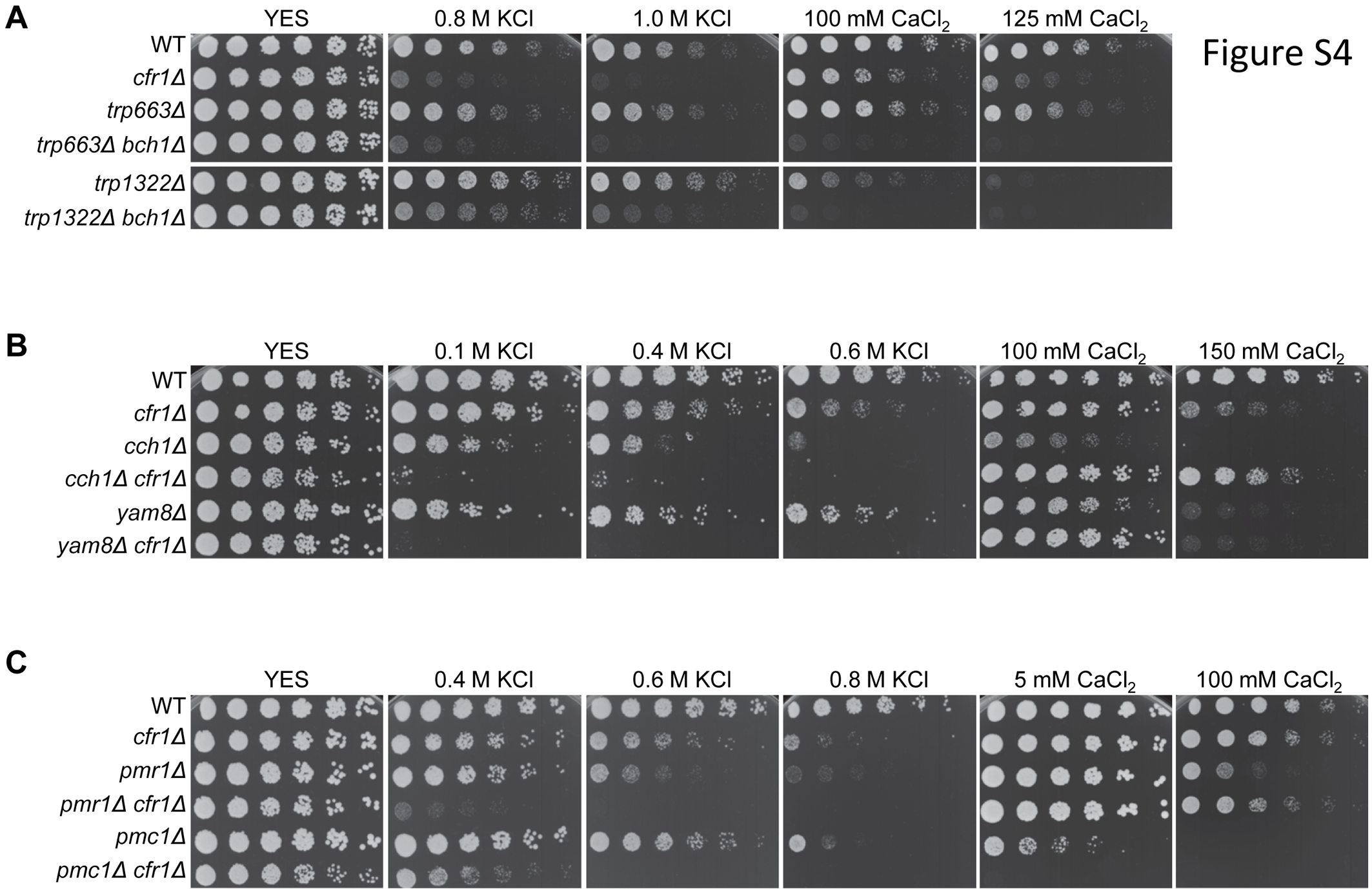

Supplement: Supplementary Figure 4 — Analysis of the relationship between exomer and calcium transporters in the presence of the indicated concentrations of potassium and calcium chlorides. (A) Genetic interaction between bch1Δ and mutants for Transient Receptor Potential (TRP) Ca2+ channels. (B) Genetic interaction between cfr1Δ and mutants for the Ca2+ ion import channels Cch1 and Yam8. (C) Genetic interaction between cfr1Δ and mutants for the plasma membrane Ca2+ P-type ATPase Pmr1 and the vacuolar Ca2+ P-type ATPase Pmc1. [file Image_4.TIF]

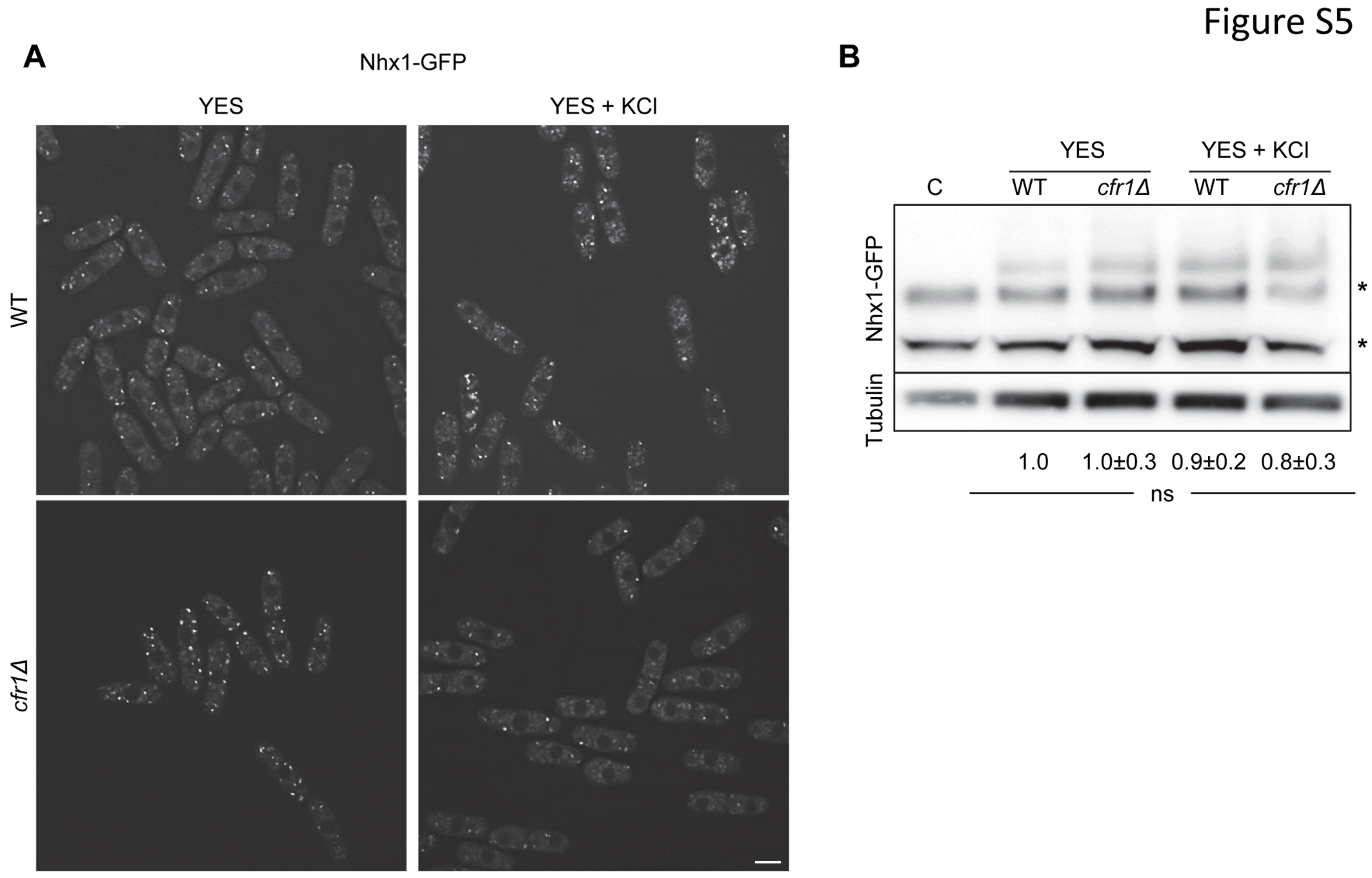

Supplement: Supplementary Figure 5 — (A) Cells from the wild-type (WT) and cfr1Δ strains bearing Nhx1-GFP and incubated in YES and YES with 0.6 M KCl for 1 h were photographed with a DeltaVision system. Images are SUM projections. Bar, 10 μm. (B) The same experimental conditions as in (A), but the cells were broken and the cell extracts were subjected to SDS-PAGE and immunoblotted with anti-GFP (upper panel) and anti-tubulin (lower panel; loading control) antibodies. Extracts from a strain that did not express Nhx1-GFP were loaded in the first lane (C) to identify unspecific bands, which are denoted by asterisks. The intensity of each Nhx1-GFP band was relativized to the value for the corresponding tubulin band, and all the values were relativized to the value for the WT grown in YES. For each value, the mean of three independent experiments, standard deviation, and statistical significance of the difference, determined using the Tukey’s multiple comparisons test are shown (ns, non-significant). [file Image_5.TIF]
