# Supplementary material for: Exomer Is Part of a Hub Where Polarized Secretion and Ionic Stress Connect
Source: Front Microbiol. 2021 Jul 19;12:708354. doi: 10.3389/fmicb.2021.708354 (PMC8326576; doi:10.3389/fmicb.2021.708354)
Supplement: Supplementary Table 1 — List of strains used in this work. [file Table_1.DOCX]

**S1 Table. List of yeast strains used in this work**

| **STRAIN** | **GENOTYPE** | **SOURCE** |
| --- | --- | --- |
| HVP30 | *leu1-32 his3-∆1 ura4-∆18 ade6 h-* | Lab stock |
| HVP117 | *leu1-32 his3-∆1 ura4-∆18 ade6 h+* | Lab stock |
| HVP241 | *cfr1::his3^+^ leu1-32 his3^?^ ura4-∆18 ade6 h+* | Lab stock |
| HVP264 | *cfr1::his3^+^ leu1-32 his3^?^ ura4-∆18 ade6 h-* | Lab stock |
| HVP2034 | *leu1^+^ his3^+^ ur4^+^ ade6^+^ h-* | Lab stock |
| HVP2092 | *apm1::ura4 leu1-32 h-* | T Kuno |
| HVP2356 | *bch1::KAN leu1-32 his3-∆1 ura4-∆18 ade6 h+* | Lab stock |
| HVP2357 | *bch1::KAN leu1-32 his3-∆1 ura4-∆18 ade6 h-* | Lab stock |
| HVP2871 | *bch1::NAT leu1-32 his3-∆1 ura4-∆18 ade6 h-* | Lab stock |
| HVP3348 | *bch1::NAT leu1-32 his3-∆1 ura4-∆18 ade6 h+* | Lab stock |
| HVP3701 | *apm3::KAN leu1-32 ura4-Δ18 ade6 h+* | Bioneer |
| HVP3953 | *HPHMX6 h-* (hph.171k) | I Hagan/ YGRC |
| HVP3954 | *gga21::KAN leu1-32 his3-∆1 ura4-∆18 ade6 h-* | Lab stock |
| HVP3956 | *gga22::KAN leu1-32 his3-∆1 ura4-∆18 ade6 h-* | Lab stock |
| HVP3958 | *tup11:.ura4^+^ tup12::ura4^+^ cta3::ura4^+^ leu1-32 ura4-∆18 ade6 h-* | Simon Whitehall |
| HVP3994 | *gga21::KAN gga22::KAN his3-Δ1 h-* | Lab stock |
| HVP3997 | *its3-1 leu1-32 ura4-∆18 h-* | PP Lab stock |
| HVP4036 | *its3-1 cfr1::his3^+^ leu1-32 his3^?^ ura4-∆18 ade6^?^ h+* | Lab stock |
| HVP4068 | *HPHMX6 leu1-32 his3-∆1 ura4-∆18 h+* | Lab stock |
| HVP4069 | *cch1::KAN leu1-32 ura4-∆18 ade6 h+* | Bioneer |
| HVP4070 | *ura4:pKK2+PCDRE-GFP his3-∆1 h+* | D Hirata/PP Lab stock |
| HVP4100 | *ura4:pKK2+PCDRE-GFP cfr1::his3^+^ leu1^?^ his3-∆1 ura4^?^ ade6^?^ h^?^* | This work |
| HVP4106 | *Pnda2:GFP-PH(GAP1):Tnda2:NAT leu1-32 his3-∆1 ura4-∆18 h+* | Lab stock |
| HVP4174 | *yam8::ura4^+^ leu1-32 ura4-∆18 ade6 h+* | Y Sanchez |
| HVP4176 | *yam8:.ura4^+^ cfr1::his3 leu1-32 his3-∆1^?^ ura4-∆18 ade6 h^?^* | Lab stock |
| HVP4178 | *cch1::KAN cfr1::his3 leu1-32 his3-∆1^?^ ura4-∆18 ade6 h^?^* | Lab stock |
| HVP4182 | *ade6:mCherry-Psy1 nmt:cta3-YFP-Flag-6HIS* | T. Nakamura/YGRC |
| HVP4438 | *nmt:cta3-YFP-Flag-6HIS cfr1::his3^+^ leu1-32^?^ his3-∆1 ura4-∆18^?^ ade6 h^?^* | This work |
| HVP4534 | *Pnda2:GFP-PH(GAP1):Tnda2:NAT cfr1::his3^+^ leu1-32 his3^?^ ura4-∆18 ade6^?^ h^?^* | Lab stock |
| HVP4243 | *Pnda2:mCherry-FYVE:Tnda2:NAT leu1-32 his3-∆1 ura4-∆18 ade6^?^ h+* | Lab stock |
| HVP4493 | *Pnda2:mCherry-FYVE:Tnda2:NAT cfr1::his3^+^ leu1-32 his3^?^ura4-∆18 ade6^?^ h+* | Lab stock |
| HVP4509 | *ent3::KAN leu1-32 his3-∆1 ura4-∆18 ade6 h+* | Bioneer |
| HVP4846 | *trk2::ura4^+^ cfr1::his3^+^ leu1-32 his3-∆1 ura4-∆18 ade6 h+* | This work |
| HVP4853 | *pmr1::ura4^+^ leu1-32 h-* | JC Ribas |
| HVP4854 | *trk2::ura4^+^ leu1-32 his3-∆1 ura4-∆18 ade6 h-* | This work |
| HVP4855 | *tup11::ura4^+^ tup12::ura4^+^ cta3::ura4^+^ bch1::NAT leu1-32 his3-∆1^?^ ura4-∆18 ade6 h-* | This work |
| HVP4872 | *cta3::ura4^+^ leu1-32 his3-∆1^?^ ura4-∆18 ade6 h-* | This work |
| HVP4874 | *cta3::ura4^+^ cfr1:.his3^+^ leu1-32 his3^?^ ura4-∆18 ade6 h-* | This work |
| HVP4875 | *pmr1::ura4^+^ cfr1::his3^+^ leu1-32 his3-∆1^?^ ura4-∆18^?^ ade6^?^ h^?^* | This work |
| HVP4894 | *nmt:cta3-YFP-Flag-6HIS leu1-32^?^ his3-∆1 ura4-∆18^?^ ade6 h^?^* | This work |
| HVP5131 | *pkd2-GFP:KAN leu1-32 his3-∆1 ura4-∆18 ade6 h-* | This work |
| HVP5133 | *pkd2-GFP:KAN cfr1::his3 leu1-32 his3-∆1 ura4-∆18 ade6 h-* | This work |
| HVP5161 | *bch1::KAN leu1^+^ his3^+^ ur4^+^ ade6^+^ h-* | This work |
| HVP5208 | *cta3-GFP:KAN leu1-32 his3-∆1 ura4-∆18 ade6 h-* | This work |
| HVP5209 | *cta3-GFP:KAN bch1::NAT leu1-32 his3-∆1 ura4-∆18 ade6 h-* | This work |
| HVP5241 | *nhx1::KAN leu1-32 his3-∆1 ura4-∆18 ade6 h-* | This work |
| HVP5249 | *nhx1::KAN cfr1::his3^+^ leu1-32 his3-∆1 ura4-∆18 ade6 h-* | This work |
| HVP5362 | *trk1::NAT trk2::ura4^+^ leu1-32 his3-∆1 ura4-∆18 ade6 h^?^* | This work |
| HVP5364 | *nhx1::KAN Pnda2:mCherry-FYVE:Tnda2:leu1^+^ leu1-32 his3-∆1 ura4-∆18 ade6 h-* | This work |
| HVP5365 | *nhx1::KAN cfr1::his3^+^ Pnda2:mCherry-FYVE:Tnda2:leu1^+^ leu1-32 his3-∆1 ura4-∆18*  *ade6 h-* | This work |
| HVP5372 | *trk1::NAT leu1-32 his3-∆1 ura4-∆18 ade6 h+* | This work |
| HVP5374 | *trk1::NAT cfr1::his3^+^ leu1-32 his3-∆1 ura4-∆18 ade6 h+* | This work |
| HVP5376 | *trk1::NAT trk2::ura4^+^ cfr1::his3^+^ leu1-32 his3-∆1 ura4-∆18 ade6 h+* | This work |
| HVP5377 | *osr2::KAN leu1-32 ura4-∆18 ade6 h+* | Bioneer |
| HVP5378 | *kha1::KAN leu1-32 ura4-∆18 ade6 h+* | Bioneer |
| HVP5380 | *osr2::KAN cfr1::his3^+^ leu1-32 his3-∆1^?^ ura4-∆18 ade6 h-* | This work |
| HVP5382 | *kha1::KAN cfr1::his3^+^ leu1-32 his3-∆1^?^ ura4-∆18 ade6 h+* | This work |
| HVP5387 | *cta3-GFP:KAN Pnda2:mCherry-FAPP1:Tnda2:NAT leu1-32 his3-∆1 ura4-∆18 ade6^?^ h+* | This work |
| HVP5389 | *cta3-GFP:KAN Pnda2:mCherry-FAPP1:Tnda2:NAT bch1::NAT leu1-32 his3-∆1 ura4-∆18 ade6^?^ h+* | This work |
| HVP5397 | *pmc1::ura4^+^ leu1-32 his3^?^ ura4-∆18 ade6^?^ h+* | J.C. Ribas |
| HVP5398 | *pkd2-GFP:KAN Pnda2:mCherry-FYVE:Tnda2:leu1^+^ leu1-32 his3-∆1^?^ ura4-∆18 ade6 h-* | This work |
| HVP5399 | *pkd2-GFP:KAN Pnda2:mCherry-FYVE:Tnda2:leu1^+^  cfr1::his3^+^ leu1-32 his3^?^*  *ura4-∆18 ade6 h-* | This work |
| HVP5400 | *cta3-GFP:KAN Pnda2:mCherry-FYVE:Tnda2:leu1^+^ leu1-32 his3-∆1 ura4-∆18 ade6 h-* | This work |
| HVP5401 | *cta3-GFP:KAN Pnda2:mCherry-FYVE:Tnda2:leu1^+^ bch1::NAT leu1-32 his3-∆1 ura4-∆18 ade6 h-* | This work |
| HVP5404 | *pkd2-GFP:KAN Pnda2:mCherry-FAPP1:Tnda2:NAT leu1-32 his3-∆1^?^ ura4-∆18 ade6^?^ h-* | This work |
| HVP5405 | *pkd2-GFP:KAN Pnda2:mCherry-FAPP1:Tnda2:NAT cfr1::his3^+^ leu1-32 his3^?^ ura4-∆18 ade6^?^ h+* | This work |
| HVP5425 | *pmc1::ura4^+^ cfr1::his3^+^ leu1-32 his3-∆1^?^ ura4-∆18 ade6^?^ h+* | This work |
| HVP5426 | *erg5::KAN leu1-32 ura4-∆18 ade6 h+* | Bioneer |
| HVP5427 | *erg28::KAN leu1-32 ura4-∆18 ade6 h+* | Bioneer |
| HVP5432 | *trp1322::ura4^+^ leu1-32 his3-∆1 h+* | Q Chen stock |
| HVP5433 | *trp663::kanMX6 leu1-32 ura4-∆18 his3-∆1 h?* | Q Chen stock |
| HVP5434 | *KanMX6-P81nmt1-pkd2 (pkd2-81KD) leu1-32 ura4-Δ18 his3-Δ1 h-* | Q Chen stock |
| HVP5436 | *HPH:GFP-trk1* *leu1-32 his3-∆1 ura4-∆18 ade6 h-* | This work |
| HVP5437 | *HPH:GFP-trk1 cfr1::his3^+^ leu1-32 his3-∆1 ura4-∆18 ade6 h-* | This work |
| HVP5446 | *erg5::KAN bch1::NAT leu1-32 ura4-∆18 ade6 h+* | This work |
| HVP5447 | *erg28::KAN bch1::NAT leu1-32 ura4-∆18 ade6 h+* | This work |
| HVP5448 | *trp663::kanMX6 bch1::NAT leu1-32 ura4-∆18 his3-∆1* | This work |
| HVP5449 | *trp1322::ura4^+^ bch1::NAT leu1-32 his3-∆1 h+ h+* | This work |
| HVP5453 | *pkd2-81KD* *bch1::NAT* *leu1-32 ura4-Δ18 his3-Δ1 h-* | This work |
| HVP5519 | *its3-GFP:KAN leu1-32 his3-∆1 ura4-∆18 ade6 h-* | This work |
| HVP5520 | *its3-GFP:KAN cfr1::his3^+^ leu1-32 his3^?^ ura4-∆18 ade6 h^?^* | This work |
| HVP5533 | *HPHMX6 Pcta3:pkd2-GFP:NAT leu1-32 his3-∆1 ura4-∆18 h+* | This work |
| HVP5534 | *HPHMX6 Pcta3:pkd2-GFP:NATcfr1::his3^+^ leu1-32 his3-∆1 ura4-∆18 h+* | This work |
| HVP5550 | *cta3-GFP:KAN Pact1^+^:mCherry:D4H:Tact1 leu1-32 his3-∆1 ura4-∆18 ade6 h-* | This work |
| HVP5551 | *cta3-GFP:KAN Pact1^+^:mCherry:D4H:Tact1 bch1::NAT leu1-32 his3-∆1 ura4-∆18*  *ade6 h-* | This work |
